# Emerging Thermosensitive Probes Based on Triamino-Phenazinium Dyes

**DOI:** 10.3390/molecules29204830

**Published:** 2024-10-12

**Authors:** Tatiana Munteanu, Frédéric Brunel, Michel Camplo, Olivier Siri

**Affiliations:** CNRS UMR 7325 Centre Interdisciplinaire de Nanoscience de Marseille (CINaM), Campus de Luminy, Aix Marseille University, 13288 Marseille, Cedex 09, France; tatiana.munteanu@univ-amu.fr (T.M.); frederic.brunel@univ-amu.fr (F.B.); michel.camplo@univ-amu.fr (M.C.)

**Keywords:** phenazinium, fluorophores, molecular thermometers

## Abstract

Temperature is an essential physical characteristic that influences all biological processes. Building on previous research on dialkylamino-functionalized rhodamine-based thermo-sensors, we investigate herein the thermosensitive properties of triamino-phenazinium dyes. Through a simple five-step synthetic route, we synthesized amino-phenazinium chromophores **6** and **7**, featuring diethylamine substituents at different positions. A comparative analysis of optical properties and thermosensitivity was conducted on these compounds and an isomer, **5**, in which butylamine moiety replaced the diethylamine group. The different emissive behaviors of the three fluorophores emphasize that not only the chemical nature but also the specific position of the alkylamine substituent play fundamental roles in the synthesis of highly emissive thermo-probes.

## 1. Introduction

Temperature is a crucial physical property that plays a role in all biological processes [1]. Fundamental curiosity along with practical emergencies have pushed the research community towards the conceptualization and development of optical nanothermometry as a reliable technique for achieving thermal sensing at the sub-micronic scale. Focusing on the study of the temperature-induced alteration of optical properties in various probes, this field of study comprises a wide variety of methods, such as infrared thermometry, Raman thermal microscopy, and luminescence nanothermometry [2,3]. Due to their superior accuracy, sensitivity, high spatial resolution and the simplicity of the corresponding experimental set-up, luminescent thermometers are frequently preferred, becoming a powerful tool for exploring different cellular processes [2,4,5,6,7]. The versatility of probes (e.g., organic dyes, organo–metallic complexes, quantum dots, polymers) as well as the various parameters related to the radiative deexcitation of luminophores (intensity, band shape, spectral position, polarization, lifetime, bandwidth) establish a dynamic playground for (bio)chemists. The majority of techniques for measuring temperature at the nanoscale rely on analyzing emission intensity. This parameter is significantly influenced by temperature changes, resulting in highly accurate measurements. Moreover, an essential advantage in measuring band intensity as a function of temperature is the facility of practical implementation and data analysis [4] as well as the ability to employ either single-band emission or ratiometric dual fluorescence [7,8].

**Figure 1:**
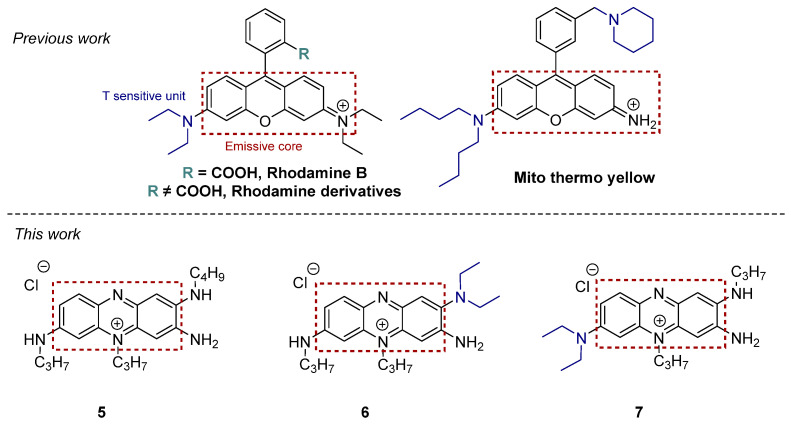
The structures of previously reported rhodamine- and rosamine-based molecular thermometers (**top**) and the phenazinium dyes described herein (**bottom**).

Small fluorescent molecules, with good cell permeability, reproductible synthesis routes, and fast responses, are among the earliest-used fluorescent sensors for thermal biology [1,9]. As a common guideline, the emission intensity of organic dyes decreases with the increase in temperature, as electrons gain thermal energy and are promoted to higher vibrational energy levels, at which the radiative deexcitation probability is reduced [2,10]. A textbook example of a temperature-sensitive fluorophore is Rhodamine B (Figure 1). This compound, belonging to the class of xanthene dyes, is well known for its bio-imaging properties and high yield of emissions, exhibiting remarkable fluorescence-quenching properties when the solution temperature is increased in the 20–80 °C interval [11]. This behavior is generally agreed to be a consequence of the presence of a diethylamino substituent, whose rotation is facilitated at higher temperatures, leading to a non-radiative relaxation to the ground state via internal conversion [12,13]. Although water solubility along with exceptional temperature sensitivity have supported the wide application of Rhodamine B as a sensor in microfluidic systems, its translation to biological environments is limited by undesired fluctuations in optical response depending on the pH or protein denaturation [14]. The organelle-targeting fluorescent molecular thermometers are particularly attractive for their potential ability to provide a detailed map of temperature variations across different cellular compartments [5]. Mitochondria-staining probes based on rosamine dyes with a temperature response in the 32–37 °C range have been reported (Mito thermo yellow, Figure 1) [15], and so too have modified rhodamines bearing thermosensitive substituents (dialkylamine, or *N*-methyl piperidine) with a certain freedom of rotation and response covering different segments of the 20–55 °C interval [16,17,18,19]. BODIPY derivatives are another class of thermosensitive probes for cells [20,21,22], but they generally suffer from several drawbacks such as relatively short emission wavelengths, high photosensitivity, and small Stokes shifts, which restrict their broader use in biological sensing and imaging applications [1,23].

Indulines, a well-established family of textile dyes structurally related to rhodamines, have been largely unexplored for biological applications, despite their widespread use in the color industry in the 19–20th century and as electrophotography agents due to their stability and versatile amino-functionalities [24,25,26,27,28,29,30]. More recently, a research study carried by our group uncovered intriguing findings regarding the significant theranostic potential of the triamino-phenazinium-chromophore-type structure **5** depicted in Figure 1. The planar, aromatic cores and cationic nature of these dyes appear to confer a preferential targeting ability and allow high-resolution imaging of mitochondria [31].

Due to our interest in exploring the versatility of triamino-phenazinium dyes and our continuous attempt to create highly fluorescent and stimuli responsive platforms, we decided to introduce a diethylamino unit to the phenazinium core, a thermosensitive group by analogy with Rhodamine B. This functional group was selectively attached to two different positions, and the resulting isomers (**6** and **7**, Figure 1), together with a derivative bearing only mono-alkylamine groups (**5** Figure 1), were comparatively analyzed in terms of their optical properties and fluorescence response to the temperature parameter. Through the study presented herein, we show that not only the nature but also the position of the alkylamine substituent play crucial roles in the synthesis of highly emissive phenazinium thermo-probes. This dual importance is clearly demonstrated by the contrasting behaviors of compounds **6** and **7**. Water solubility, excellent emission efficiency (a 68% quantum yield), and the high thermosensitivity of triamino-phenazinium **7** (35% loss of emission intensity) make this derivative a reasonable choice for further biological testing.

## 2. Results

### 2.1. Synthesis and Characterization of Phenazinium Dyes

**Scheme 1:**
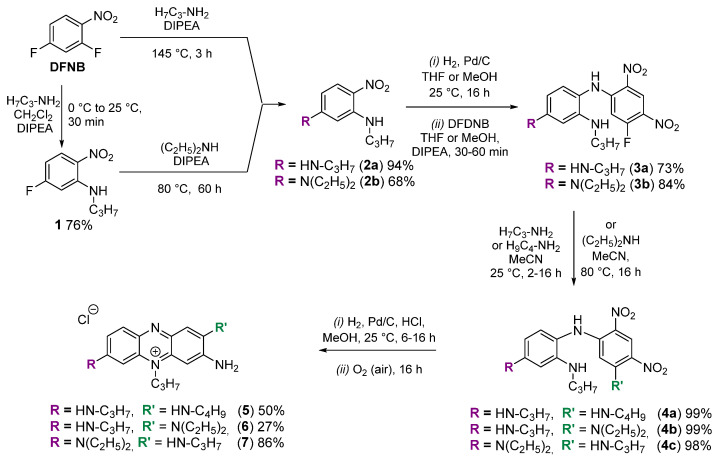
Synthesis of phenazinium dyes **5**–**7**.

The synthesis of the three phenazinium dyes is illustrated in Scheme 1 and was performed according to a previously reported protocol [31]. Starting from the commercially available 2,4-difluoronitrobenzene, in a simple one- or two-step nucleophilic substitution with propylamine or diethylamine, we obtained the diamino-benzene precursors **2a** and **2b** in good to excellent yields. Next, the catalytic reduction of the nitro function was carried out, leading to intermediates **3a** and **3b**, obtained as brown solids in very good yields. For the fluorine substitution performed afterward, we slightly modified the protocol previously employed for **4a** type of intermediates, which involved the use of Hünig’s base. Here, we show that precursors **4a**−**c** could be synthesized in quantitative yields without the need for *N*,*N*-diisopropylethylamine since the propylamine, butylamine or the diethylamine act as both a nucleophile and a base. Finally, the reduction of the two nitro moieties and the oxidative cyclization of the fully reduced species (non-isolated) led to the formation of the expected phenazinium **5**–**7** in yields spanning from 27% to 86%.

The synthesized dyes were characterized using HRMS, with the bulk purity confirmed via ^1^H and ^13^C NMR. Additionally, the structures of derivatives **6** and **7** were fully established via single-crystal X-ray diffraction. Single crystals of **6** were obtained by the slow evaporation (at room temperature) of a dichloromethane (DCM) solution, in which a few drops of methanol were added. In case of compound **7**, the crystals were formed by the slow diffusion of pentane in a chloroform solution of **7**, kept at 4 °C.

**Figure 2:**
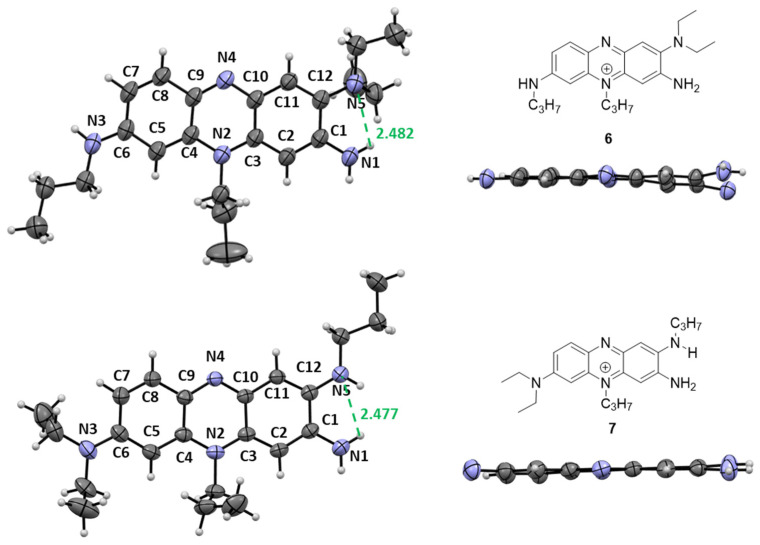
ORTEP views of **6** (**top left**: top view, **top right**: side view) and **7** (**bottom left**: top view, **bottom right**: side view). Ellipsoids were drawn at 50% probability. Counterions, alkyl chains for the side views in **6** and **7**, and the co-crystallized solvent molecules in **7** were omitted for clarity. Selected bond lengths (Å) for **6**: N(1)-C(1) 1.339(2), N(2)-C(3) 1.373(2), N(2)-C(4) 1.384(2), N(3)-C(6) 1.343(3), N(4)-C(9) 1.338(3), N(4)-C(10) 1.334(2), N(5)-C(12) 1.395(2), C(1)-C(2) 1.391(3), C(1)-C(12) 1.451(3), C(2)-C(3) 1.388(3), C(3)-C(10) 1.432(3), C(4)-C(5) 1.391(3), C(4)-C(9) 1.428(3), C(5)-C(6) 1.399(3), C(6)-C(7) 1.431(3), C(7)-C(8) 1.344(3), C(8)-C(9) 1.426(2), C(10)-C(11) 1.418(3), C(11)-C(12) 1.369(3). Selected bond lengths (Å) for **7**: N(1)-C(1) 1.338(3), N(2)-C(3) 1.371(3), N(2)-C(4) 1.387(3), N(3)-C(6) 1.360(3), N(4)-C(9) 1.344(3), N(4)-C(10) 1.330(3), N(5)-C(12) 1.356(3), C(1)-C(2) 1.392(3), C(1)-C(12) 1.460(3), C(2)-C(3) 1.386(3), C(3)-C(10) 1.449(3), C(4)-C(5) 1.394(3), C(4)-C(9) 1.423(3), C(5)-C(6) 1.398(3), C(6)-C(7) 1.432(3), C(7)-C(8) 1.353(3), C(8)-C(9) 1.414(3), C(10)-C(11) 1.412(3), and C(11)-C(12) 1.373(3).

The molecular structures are shown in Figure 2. Both **6** and **7** crystallized in the triclinic P-1 space group. While the asymmetric unit of **6** contains one molecule, the structural elucidation of **7** unveils two independent units co-crystallized with one molecule of water and two molecules of chloroform (ESI Figure S21). Analysis of the bond lengths and angles of the two dyes confirmed the rigid, tricyclic phenazinium cationic core, with chloride serving as a counterion. Examination of the bond distances within the N(1)–C(1)–C(2)–C(3)–N(2)-C(4)-C(5)-C(6)-N(3) segment shows a full delocalization of the conjugated π system, whereas the “upper part”, i.e., N(5)–C(12)–C(11)–C(10)–N(4)-C(9)-C(8)-C(7), consists of a succession of single and double bonds, implying poorer π delocalization. Additionally, the single-bond character of the C-C bonds between the two segments, C(1)-C(12) in particular, indicates a decrease in the aromaticity of the tricyclic phenazinium core with the stabilization of the cationic charge. The packing motif of **6** reveals a pseudo-dimer unit, stabilized by weak π-π interactions, with a distance of 3.418 Å between the two units (ESI Figure S31). The two molecules are parallelly arranged in a head-to-tail fashion to minimize the positive charge repulsion. In the solid state, dye **7** is packed in a less organized fashion (ESI Figures S32 and S33), with the distances between the molecules (3.495 Å) falling in the range of weak π-π interaction.

The square plane calculated throughout the 14 atoms of the phenazinium core indicates that the skeleton is planar, with a maximum deviation from planarity for **6** determined to be 0.149 Å and, for **7**, 0.057 Å. These differences indicate a more rigid structure for derivative **7** and could therefore help explain the remarkable increase in emission efficiency when switching from **6** to **7**. The hydrogen bond between the N(5) and the primary amine group, depicted in green in Figure 2, points towards greater freedom of movement for the diethylamine in derivative **7**, leading to a gain in thermosensitivity for the latter (vide infra Section 2.2).

### 2.2. Optical Properties

**Figure 3:**
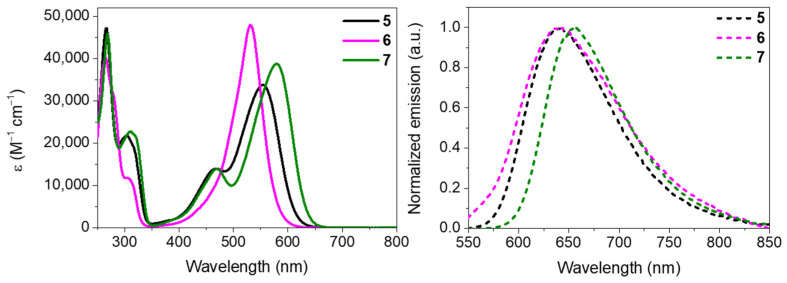
Electronic absorption (**left**) and normalized emission (**right**) of dyes **5**–**7** in acetonitrile (λ_ex_ = 530 nm).

The optical properties of the structural isomers **5**, **6**, and **7** were ascertained in acetonitrile (MeCN), dimethylformamide (DMF), and water (H_2_O) at room temperature (Figure 3, Table 1, and ESI Figures S34–S36). The three phenazinium dyes absorb light in the green–yellow region of the electromagnetic spectrum, with maximums centered at 552 nm for **5** and 531 nm for **6** and slightly red-shifted for **7**, with a band at 579 nm. For **5** and **7**, the absorption band presents a shoulder on the high-energy side, which is absent in the case of isomer **6**. The absorption is distinguished by high molar extinction coefficients in the 10 ^4^ M^−1^ cm^−1^ region (between 33,000 and 48,000 M^−1^ cm^−1^), quite characteristic of triamino-phenazinum chromophores [31]. The emission profiles of **5**–**7** exhibit a typical, rather wide, featureless fluorescence band in the red region, peaking at approx. 640–650 nm, with an important shift towards the far-red end as acetonitrile is replaced by water. In general, due to their rigid structures, phenazinium dyes are highly emissive, which was further confirmed by the determined quantum yields of 58–61% for **5** and 67–68% for **7**. Unexpectedly, the fluorescence of derivative **6** was considerably quenched, with a quantum yield of only 0.6%. In an attempt to explain the very low values of quantum yield for **6**, we turned to a previous study we reported [31], in which it was shown that upon the deprotonation of a closely related phenazinium dye, a very poorly emissive quinone was generated. We therefore believe that at very high dilutions, specific for spectroscopy measurements (10^−5^–10^−6^ M), dye **6** becomes vulnerable to deprotonation by water traces and converts into a quinone [**6**–H^+^] (ESI Scheme S1). This specific behavior might be due to the presence of a diethylamine unit, which impacts the acidic character of the NH_2_ protons in the ortho position. To confirm this hypothesis, we further performed a protonation study (ESI Figure S42), the results of which point towards the formation of a new species, with a considerably improved quantum yield of 34%, as expected for **6**.

The calculated Stokes shifts for **5**–**6** returned nontrivial values in the range of 2000–3000 cm^−1^. Together with high emission efficiency, this parameter is crucial for the design of biological markers with minimized spectral overlap between absorption and emission, which attenuates the self-quenching phenomena and amplifies the signal-to-background ratio [32].

### 2.3. Temperature Sensitivity

**Figure 4:**
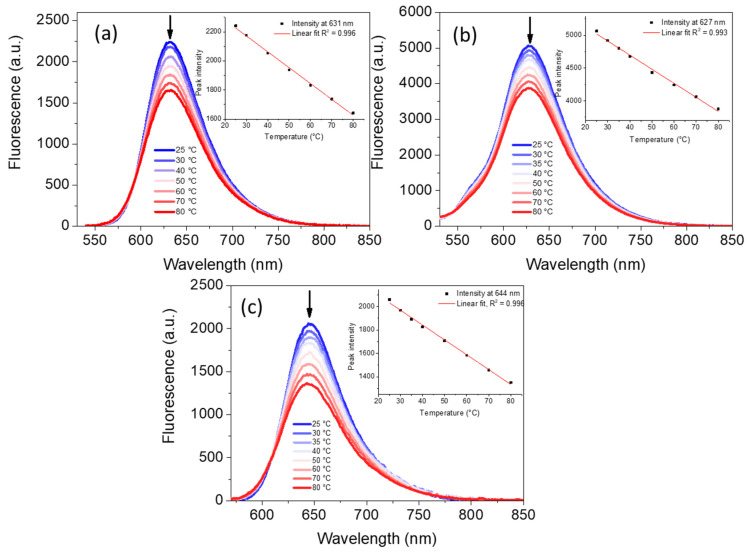
Fluorescence spectra of **5**, **6**, and **7** in DMF while being gradually heated from 25 °C to 80 °C (**a**, **b** and **c**, respectively). λ_ex_ = 530 nm for **5**, λ_ex_ = 515 nm for **6**, and λ_ex_ = 565 nm for **7**. The inset shows the linear relationship between the intensity of the emission (taken as the maximum value of the peak) and temperature.

The thermosensitivity of the three fluorophores was studied in DMF because the higher boiling point allowed a wider range of measurements (from 25 °C to 80 °C), minimizing the erroneous results from solvent evaporation. The fluorescence spectra of **5**–**7** as a function of temperature are depicted in Figure 4. All three dyes respond to temperature, as displayed by the gradual loss in fluorescence intensity with heating. Derivative **7**, nevertheless, seems to have a more advantageous sensitivity, with a 35% loss of signal intensity, which can be compared to the 27% loss in the case of **5** and the 24% loss calculated for **6**. A linear relationship was found between the value of the of the emission peak (λ_max_) and the temperature (expressed in °C), with very good fitting for **6** (R^2^ = 0.993) and excellent fitting for **5** and **7** (R^2^ = 0.996). Additionally, the thermosensitivity of the three dyes was determined in DMSO, highlighting similar behavior, with linear correlations found for **5** and **7** and a second-degree polynomial fitting for compound **6** (ESI Figures S39–S41). The loss of emission intensity in DMSO was calculated to be 26% for both **5** and **6** and 31% for derivative **7**.

## 3. Discussion

In this study, we tested a hypothesis, derived from literature research, that highlights the thermosensitive properties of dialkylamino-functionalized rhodamines by translating it to the emerging family of triamino-phenazinium dyes. Using a simple pathway and starting from a commercially available building block, we synthesized the phenazinium chromophores **6** and **7**, bearing diethylamine substituents at the C12 and C6 positions, respectively (see the X-ray structures in Figure 2), and a monoalkylamine-funcionalized isomer **5**, bearing a butylamine group instead. The three dyes were fully characterized via NMR and HMRS as well as single-crystal X-ray techniques for **6** and **7**. Furthermore, a comparative analysis of the optical properties and thermosensitivity of the dyes was performed. While the absorption profiles did not reveal important differences between the three compounds, fluorescence measurements indicated an important loss of emission efficiency in derivative **6**, in which the diethylamine group is linked to the C12 atom. A possible explanation, derived from the analyzed single-crystal XRD structure, lies in the slightly less rigid structure of **6** compared to **7** as well as an “unfavorable” position of the diethylamino substituent when introduced at the C12 position. The evaluation of the fluorescence response to temperature unveiled a good return for all three dyes, with a decrease in emission intensity upon heating, pointing towards the nontrivial thermosensitivity of the mono-alkylamine substituents as well. Small-molecule dyes display temperature-dependent fluorescence primarily due to the rotational and vibrational freedom of their substituents, leading to nonradiative excited-state decay that can be significantly affected by temperature. The diethylamino group is recognized for inducing such temperature sensitivity, largely because of its ability to rotate freely. We propose that this same principle can be extended to monoalkylamino chains as well, thus explaining the observed important thermosensitivity of molecule **5**. However, we found that derivative **7** stands out in terms of both emission efficiency (68% quantum yield) and the highest loss in fluorescence intensity when gradually increasing the temperature from 25 °C to 80 °C (35% loss) as a consequence of a non-hindered rotation of the diethylamine moiety.

In this study, we developed a new family of thermosensitive probes based on triamino-phenazinium dyes **5**–**7** that could be prepared easily and in just a few steps thanks to the versatility of the synthesis method used. Through a judicious design, i.e., a rigid cationic phenazinium core prompting radiative deexcitation; short alkylamine chains; and the selective introduction of a diethylamine moiety at the C6 position to enhance thermosensitivity, we highlighted that dye **7** (with a 35% loss of emission intensity at 80 °C) possesses promising features for bio-sensing applications. Testing in a biological medium is now envisaged, as we have previously shown that triamino-phenazinium dyes preferentially stain mitochondria organelles [31].

## 4. Materials and Methods

### 4.1. General Remarks and Analysis Conditions

**Reagents**. All reagents and solvents were purchased from Alfa-Aesar (Haverhill, MA, USA) or Sigma-Aldrich (St. Louis, MO, USA)and used as received. When heating was required, oil baths were used. Column chromatography was performed using silica gel (60–120 mesh) and alumina 90 neutral (63–200 μm, Beckmann grade I). Analytical thin-layer chromatography (TLC) was performed on precoated silica gel-60 F254 (0.5 mm) aluminum plate or precoated Al_2_O_3_ gel-60 neutral (0.2 mm) aluminum plate. Visualization of the spots on TLC plates was achieved via exposure to UV light. Filter aid was performed using Celite AW standard Supercel or Celite type 545. Unless otherwise specified, the desired product was dried under vacuum (<10 mbar) for over 5 h at room temperature.

**Analytical Methods and Apparatus**. NMR spectra were recorded on a JEOL ECS400 spectrometer (JEOL, Tokyo, Japan) operating at 400 MHz for ^1^H and 100 MHz for ^13^C, respectively, at room temperature. NMR chemical shifts are given in ppm (δ) relative to Me_4_Si, with solvent resonances used as internal standards (CDCl_3_: 7.26 ppm for ^1^H and 77.2 for ^13^C; CD_3_OD: 3.31 ppm for ^1^H and 49.0 for ^13^C). The multiplicity of signals is designated by the following abbreviations: s, singlet; br s, broad singlet; d, doublet; t, triplet; q, quartet; quint, quintet; sext, sextet; m, multiplet; br m, broad multiplet. Coupling constants, J, are reported in Hertz (Hz). High-resolution mass spectrometry (HRMS) analysis was performed using the “Spectropole” of Aix-Marseille University.

**Crystallography**. Crystals were mounted on a Rigaku Oxford Diffraction SuperNova diffractometer and measured at 293 K using Cu radiation (λ = 1.54184 Å). Data collection, reduction, and multiscan ABSPACK correction were performed with CrysAlisPro version 1.171.43.125 (Rigaku Oxford Diffraction, Tokyo, Japan). Using Olex2 [33], the structures were solved with the ShelXT [34] structure solution program using Intrinsic Phasing and refined with ShelXL [34] using least-squares minimization.

Fluorescence. Emission spectra were measured using a Horiba-Jobin Yvon Fluorolog-3 spectrofluorometer (Horiba, Kyoto, Japan) equipped with a three-slit double-grating excitation and a spectrograph emission monochromator with dispersions of 2.1 nm mm^−1^ (1200 grooves per mm). A 450 W xenon continuous-wave lamp provided excitation. The luminescence of diluted solutions was detected at right angles using 10 mm quartz cuvettes.

Fluorescence quantum yields Φ were measured in diluted solutions with an optical density lower than 0.1 using the following equation:

(1) $$\frac{\Phi_{x}}{\Phi_{r}}=\left(\frac{A_{r}(\lambda )}{A_{x}(\lambda )}\right)\left(\frac{n_{x}^{2}}{n_{r}^{2}}\right)\left(\frac{D_{x}}{D_{r}}\right)$$

where *A* is the absorbance at the excitation wavelength (*λ*), *n* is the refractive index, and *D* is the integrated intensity. “*r*” and “*x*” stand for reference and sample. The fluorescence quantum yields were measured with rhodamine B as a reference (Φ = 70% in MeOH, and λ_ex_ = 530 nm or, for **6** in acidic acetonitrile, λ_ex_ = 510 nm) [35].

The spectra of fluorescence as a variation of temperature were recorded using a JASCO Spectrofluorometer FP-8600 (JASCO, Tokyo, Japan) equipped with a Peltier JASCO ETC 815 (JASCO, Tokyo, Japan).

### 4.2. Synthesis Protocols and Characterization

Compound **1**. To a stirred solution of 2,4-difluoronitrobenzene (2 mL, 18.23 mmol, 1 equiv.) in dichloromethane (50 mL) at 0 °C was added 1-propylamine (1.5 mL, 18.23 mmol, 1 equiv.) and then *N*,*N*-diisopropylethylamine (3.49 mL, 20 mmol, 1.1 equiv.). The reaction mixture was allowed to warm up to room temperature (over 30 min), after which time the reaction was complete (monitored via TLC). The solvent was evaporated in vacuo to afford yellow residue that was purified over silica gel column gel to afford the desired product as a yellow oil (2.74 g, 13.8 mmol, 76%).

R_f_: 0.5 (petroleum ether/ethyl acetate, 96:4). ^1^H NMR (400 MHz, CDCl_3_): δ = 8.17 (m, 2H), 6.47 (dd, *J* = 11.6 Hz, *J* = 2.4 Hz, 1H), 6.35 (ddd, *J* = 9.6 Hz, 4.8 Hz, 2.4 Hz, 1H), 3.22 (td, *J* = 7 Hz, *J* = 5.2 Hz, 2H), 1.76 (sext, *J* = 7.3 Hz, 2H), 1.06 (t, *J* = 7.4 Hz, 3H). ^13^C NMR (101 MHz, CDCl_3_): δ = 167.7 (d, *J*^1^_C–F_ = 257 Hz, C), 147.7 (d, *J*^3^_C–F_ = 14 Hz, C), 130.1 (d, *J*^3^_C–F_ = 13 Hz, CH), 128.8 (C), 103.9 (d, *J*^2^_C–F_ = 25 Hz, CH), 99.3 (d, *J*^2^_C–F_ = 27 Hz, CH), 45.1 (CH_2_), 22.1 (CH_2_), 11.6 (CH_3_). ^19^F NMR (376 MHz, CDCl_3_): δ 99.40 (s).

Compound **2a**. To a pressure bomb were introduced 2,4-difluoronitrobenzene (2 mL, 18.23 mmol, 1 equiv.), 1-propylamine (6.14 mL, 74.7 mmol, 4.1 equiv.), and *N*,*N*-diisopropylethylamine (5.4 mL, 31 mmol, 1.7 equiv.). The bomb was closed with a Teflon seal. The mixture was heated to 145 °C for 3 h. After the mixture had cooled to room temperature, ethanol (5 mL) was added. This suspension was scatted with ultrasound. The resulting solid in the suspension was isolated via filtration, rinsed with hot water, and dried under vacuum to afford the desired product as a yellow powder (4.07 g, 17.14 mmol, 94%).

^1^H NMR (400 MHz, CDCl_3_): δ = 8.53 (br s, 1H), 8.01 (d, *J* = 8.8 Hz, 1H), 5.90 (dd, *J* = 9.2 Hz, 2 Hz, 1H), 5.63 (d, *J* = 2 Hz, 1H), 4.46 (br s, 1H), 3.18 (m, 4H), 1.75 (sext, *J* = 7.2 Hz, 2H), 1.68 (sext, *J* = 7.6 Hz, 2H), 1.05 (t, *J* = 7.4 Hz, 3H), 1.02 (t, *J* = 7.4 Hz, 3H). ^13^C NMR (101 MHz, CDCl_3_): δ = 154.5 (C), 148.7 (C), 129.3 (CH), 123.6 (C), 104.8 (CH), 89.2 (CH), 45.1 (CH_2_), 44.8 (CH_2_), 22.4 (CH_2_), 22.1 (CH_2_), 11.8 (CH_3_), 11.6 (CH_3_).

Compound **2b**. To a round-bottom flask equipped with a condenser were introduced 825 mg of precursor **1** (4.16 mmol, 1 equiv.), 4.3 mL of diethylamine (41.6 mmol, 10 equiv.), and 7.2 mL of *N*,*N*-diisopropylethylamine (41.6 mmol, 10 equiv.). This mixture was stirred at 80 °C for 60 h. The reaction was left to cool down. The solvent evaporated, and the residue was purified on a silica gel column using petroleum ether/ethyl acetate (96:4) as an eluent to give 717 mg of yellow oily solid (2.83 mmol, 68% yield).

R_f_: 0.2 (petroleum ether/ethyl acetate, 96:4). ^1^H NMR (400 MHz, CDCl_3_): δ = 8.46 (br s, 1H, NH), 8.06 (d, *J*^3^ = 9.7 Hz, 1H, CH), 6.08 (dd, *J*^3^ = 9.7 Hz, *J*^4^ = 2.4 Hz, 1H, CH), 5.64 (d, *J*^4^= 2.2 Hz, 1H, CH), 3.45 (q, *J*^3^ = 7 Hz, 4H, CH_2_), 3.23 (q, *J*^3^ = 7 Hz, 2H, CH_2_), 1.79 (sext, *J*^3^ = 7.3 Hz, 2H, CH_2_), 1.25 (t, *J*^3^ = 7 Hz, 6H, CH_3_), 1.07 (t, *J*^3^ = 7.7 Hz, 3H, CH_3_).^13^C NMR (101 MHz, CDCl_3_): δ = 153.3 (C), 148.2 (C), 129.5 (CH), 122.9 (C), 102.9 (CH), 90.0 (CH), 45.0 (CH_2_), 44.7 (CH_2_), 22.2 (CH_2_), 12.8 (CH_3_), 11.8 (CH_3_). HRMS (ESI+) calculated for [C^+^]: 252.1707 (C_13_H_22_N_3_O_2_^+^) found: 252.1707.

Compound **3a**. A solution of **2a** (1.5 g, 6.3 mmol, 1 equiv.) in 60 mL of tetrahydrofuran was hydrogenated (*p* = 40 bars) in the presence of Pd/C (5% wt, % 2 mol, 270 mg) overnight. After reducing the pressure, the solution was degassed by bubbling argon in the mixture and keeping it at 0 °C. 1,5-Difluoro-2,4-dinitrobenzene (1.22 g, 6 mmol, 0.95 equiv.) was added at 0 °C while stirring, and the mixture was allowed to warm up to room temperature (over 60 min). Pd/C was then removed via filtration through a Celite^®^ plug. The crude product was purified on silica gel column chromatography, using dichloromethane as an eluent, and then over a silica gel plug, with petroleum ether/ethyl acetate (9:1) as an eluent, to afford the desired product as a brown-reddish solid (1.8 g, 4.6 mmol, 73% yield).

R_f_: 0.5 (dichloromethane/petroleum ether, 6:4). ^1^H NMR (400 MHz, CDCl_3_): δ = 9.31 (s, 1H), 9.15 (d, *J* = 8 Hz, 1H), 6.84 (d, *J* = 8.4 Hz, 1H), 6.55 (d, *J* = 13.6 Hz, 1H), 6.02 (dd, *J* = 8.4 Hz, 2.4 Hz, 1H), 5.97 (d, *J* = 2.4 Hz, 1H), 3.80 (br s, 1H), 3.70 (br s, 1H), 3.12 (t, *J* = 7 Hz, 2H), 3.09 (m, 2H), 1.69 (sext, *J* = 7.2 Hz, 2H), 1.59 (sext, *J* = 7.2 Hz, 2H), 1.03 (t, *J* = 7 Hz, 3H), 0.92 (t, *J* = 7.4 Hz, 3H). ^13^C NMR (101 MHz, CDCl_3_): δ = 161.3 (d, *J*^1^_C–F_ = 272 Hz, C), 150.6 (d, *J*^2^_C–F_ = 13 Hz, C), 150.3 (C), 145.2 (C), 129.6 (CH), 127.8 (CH), 127.0 (C), 126.9 (C), 110.8 (C), 104.1 (d, *J*^2^_C–F_ = 27 Hz, CH), 101.7 (CH), 95.2 (CH), 45.8 (CH_2_), 45.3 (CH_2_), 22.8 (CH_2_), 22.6 (CH_2_), 11.8 (CH_3_), 11.7 (CH_3_). ^19^F NMR (376 MHz, CDCl_3_): δ = −105.35 (s).

Compound **3b**. A solution of **2b** (700 mg, 2.78 mmol, 1 equiv.) in 60 mL of methanol was hydrogenated (*p* = 40 bars) in the presence of Pd/C (5% wt, % 2 mol, 118 mg) overnight. After reducing the pressure, the solution was degassed by bubbling Argon in the mixture and keeping it at 0 °C. 1,5-Difluoro-2,4-dinitrobenzene (539 mg, 2.64 mmol, 0.95 equiv.) was added at 0 °C while stirring, and the mixture was allowed to warm up to room temperature (over 60 min). Pd/C was then removed via filtration through a Celite^®^ plug. The crude product was purified on silica gel column chromatography, using dichloromethane as an eluent, and then over a silica gel plug, with petroleum ether/ethyl acetate (9:1) as an eluent, to afford the desired product as a brown-reddish solid (945 mg, 2.33 mmol, 84% yield).

R_f_: 0.7 (dichloromethane); 0.2 (petroleum ether/ethyl acetate, 9:1). ^1^H NMR (400 MHz, CDCl_3_): δ = 9.35 (s, 1H, NH), 9.10 (d, *J*^3^ = 7.7 Hz, 1H, CH), 6.88 (d, *J*^3^ = 8.6 Hz, 1H, CH), 6.60 (d, *J*^3^_H–F_ = 13.5 Hz, 1H, CH), 6.09 (dd, *J*^3^ = 8.6 Hz, *J*^4^ = 2.1 Hz, 1H, CH), 6.01 (d, *J*^4^_H–F_ = 2.4 Hz, 1H, CH), 3.78 (br s, 1H, NH), 3.42 (q, *J*^3^ = 6.8 Hz, 4H, CH_2_), 3.13 (t, *J*^3^ = 6.1 Hz, 2H, CH_2_), 1.65 (sext, *J*^3^ = 7.3 Hz, 2H, CH_2_), 1.24 (t, *J*^3^ = 7.2 Hz, 6H, CH_3_), 0.97 (t, *J*^3^ = 7.2 Hz, 3H, CH_3_). ^13^C NMR (101 MHz, CDCl_3_): δ = 161.1 (d, *J*^1^_C–F_ = 271 Hz, C), 150.6 (d, *J*^2^_C–F_ = 13 Hz, C), 149.2 (C), 145.0 (C), 128.3 (CH), 127.6 (CH), 126.7 (C), 126.6 (C), 109.4 (C), 104.0 (d, *J*^2^_C–F_ = 27.8 Hz, CH), 100.9 (CH), 94.2 (CH), 45.2 (CH_2_), 44.5 (CH_2_), 22.5 (CH_2_), 12.7 (CH_3_), 11.6 (CH_3_). ^19^F NMR (376 MHz, CDCl_3_): δ = −105.73 (s). HRMS (ESI+) calculated for [M+H^+^]: 406.1885 (C_19_H_25_FN_5_O_4_^+^) found: 406.1884.

Compound **4a**. In a two-neck flask, 350 mg of **3a** (0.89 mmol, 1 equiv.) was dissolved in 15 mL of acetonitrile, and 265 µL of butylamine (3.1 mmol, 3 equiv.) was added. The solution was stirred at room temperature for 2 h. After the evaporation of the solvent, hot ethanol (10 mL) was added to the crude residue. An asphalt-like resulting material was collected via filtration and washed with hot water (30 mL); it was then recovered via dissolution in dichloromethane, dried over anhydrous MgSO_4_, and filtered. After the solvent was removed under reduced pressure and the solid was dried under vacuum, the desired product was obtained as a dark asphalt-like solid (396 mg, 0.88 mmol, 99% yield).

^1^H NMR (CDCl_3_, 400 MHz): δ = δ = 9.27 (s, 1H), 9.12 (s, 1H), 8.21 (t, *J* = 5.2 Hz, 1H), 6.87 (d, *J* = 8.4 Hz, 1H), 6.01 (dd, *J* = 8 Hz, 2.4 Hz, 1H), 5.97 (d, *J* = 2 Hz, 1H), 5.70 (s, 1H), 3.81 (t, *J* = 5.2 Hz, 1H), 3.71 (br s, 1H), 3.11 (t, *J* = 7.2 Hz, 2H), 3.07 (m, 2H), 3.02 (m, 2H), 1.67 (sext, *J* = 7.2 Hz, 2H), 1.58 (m, 2H), 1.52 (m, 2H), 1.33 (sext, *J* = 7.4 Hz, 2H), 1.03 (t, *J* = 7.6 Hz, 3H), 0.92 (t, *J* = 7.4 Hz, 3H), 0.89 (t, *J* = 7.4 Hz, 3H). ^13^C NMR (CDCl_3_, 101 MHz): δ = 149.7 (C), 149.2 (C), 148.6 (C), 145.3 (C), 129.6 (C), 128.8 (CH), 125.0 (CH), 124.5 (C), 112.1 (C), 101.6 (CH), 95.3 (CH), 93.1 (CH), 46.0 (CH_2_), 45.4 (CH_2_), 42.8 (CH_2_), 30.3 (CH_2_), 22.9 (CH_2_), 22.7 (CH_2_), 20.2 (CH_2_), 13.7 (CH_3_), 11.8 (CH_3_), 11.7 (CH_3_).

Compound **4b**. In a two-neck flask, 200 mg of **3a** (0.51 mmol, 1 equiv.) was dissolved in 15 mL of acetonitrile, and 158 µL of diethylamine (1.53 mmol, 3 equiv.) was added. The solution was stirred at reflux for 16 h. After the reaction was stopped, it was concentrated under low pressure, and the residue was extracted in dichloromethane, washed with 100 mL of distilled water, and extracted with 100 mL of dichloromethane. The organic layers were collected, dried over MgSO_4_, and filtered, and the solvent was evaporated. The obtained residue was purified over a silica gel plug with dichloromethane, and after solvent evaporation, it yielded 223 mg of brown solid (0.5 mmol, 99% yield).

^1^H NMR (400 MHz, CDCl_3_): δ = 9.12 (s, 1H, CH), 8.80 (d, *J*^4^ = 2.18 Hz, 1H, CH), 6.87 (d, *J*^3^ = 8.2 Hz, 1H, CH), 6.01 (d, *J*^3^ = 8.3 Hz, 1H, CH), 5.97 (s, 1H, NH), 5.94 (s, 1H, CH), 3.83 (br s, 2H, NH), 3.12 (m, 8H, CH_2_), 1.70 (q, *J*^3^ = 7.2 Hz, 2H, CH_2_), 1.61 (q, *J*^3^ = 7.2 Hz, 2H, CH_2_), 1.08 (m, 9, CH_3_), 0.93 (t, *J*^3^ = 7.4 Hz, 3H, CH_3_). ^13^C NMR (101 MHz, CDCl_3_): δ = 149.5 (C), 149.4 (C), 148.2 (C), 145.4 (C), 131.1 (C), 128.8 (CH), 128.6 (CH), 123.6 (C), 112.2 (C), 101.5 (CH), 100.8 (CH), 95.3 (CH), 46.0 (CH_2_), 45.9 (2CH_2_), 45.3 (CH_2_), 22.8 (CH_2_), 22.6 (CH_2_), 12.0 (2CH_3_), 11.7 (CH_3_), 11.6 (CH_3_). HRMS (ESI+) calculated for [C^+^]: 445.2558 (C_22_H_33_N_6_O_4_^+^) found: 445.2554.

Compound **4c**. In a two-neck flask, 280 mg of **3b** (0.7 mmol, 1 equiv.) was dissolved in 25 mL of acetonitrile, and 230 µL of 1-propylamine (2.8 mmol, 4 equiv.) was added. The solution was stirred at room temperature for 16 h. After evaporating the solvent, the residue was aliquoted in 100 mL of deionized water, ultrasonicated, and filtered under vacuum. The precipitate was washed with another 100 mL of deionized water and quickly with cold methanol, yielding 306 mg of orange product (0.68 mmol, 98% yield).

^1^H NMR (400 MHz, CDCl_3_): δ = 9.27 (s, 1H, NH), 9.14 (s, 1H, CH), 8.22 (br s, 1H, NH), 6.91 (d, *J*^3^ = 8.8 Hz, 1H, CH), 6.10 (dd, *J*^3^ = 8.8 Hz, *J*^4^ = 2.1 Hz, 1H, CH), 6.02 (d, *J*^4^ = 2.1 Hz, 1H, CH), 5.73 (s, 1H, CH), 3.78 (br s, NH), 3.41 (q, *J*^3^ = 6.8 Hz, 4H, CH_2_), 3.09 (br m, 2H, CH_2_), 3.03 (q, *J*^3^ = 6.5 Hz, 4H, CH_2_), 1.64 (m, 4H, CH_2_), 1.22 (t, *J*^3^ = 6.8 Hz, 6H, CH_3_), 0.95 (m, 6H, CH_3_). ^13^C NMR (101 MHz, CDCl_3_): δ = 149.2 (C), 148.8 (C), 148.6 (C), 145.2 (C), 129.6 (CH), 128.5 (CH), 125.0 (C), 124.5 (C), 110.8 (C), 101.2 (CH), 94.6 (CH), 93.2 (CH), 45.4 (CH_2_), 44.8 (CH_2_), 44.7 (CH_2_), 22.7 (CH_2_), 21.6 (CH_2_), 12.8 (CH_3_), 11.8 (CH_3_), 11.5 (CH_3_). HRMS (ESI+) calculated for [M + H^+^]: 445.2558 (C_22_H_33_N_6_O_4_^+^) found: 445.2554.

Compound **5**. A solution of **4a** (344 mg, 0.77 mmol, 1 equiv.) in methanol (40 mL) was hydrogenated (30 bars) in the presence of Pd/C (5 wt. %5 mol, 82 mg) and HCl (12 M, 0.4 mL) for 16 h. Then, the mixture was stirred under air for another 16 h. Pd/C was removed via filtration through a Celite^®^ plug, which was rinsed multiple times with methanol and dichloromethane. After removal of the solvent under reduced pressure, the resulting solid was purified via an alumina oxide gel chromatography column (neutral, activity I, eluent dichloromethane/methanol, 95:5, solid deposition). The pink fraction was collected, concentrated under vacuum, and repassed on the same type of chromatography column to afford 152 mg of product (0.38 mmol, 50% yield).

R_f_: 0.5 (Al_2_O_3_, dichloromethane/methanol, 95:5). ^1^H NMR (400 MHz, CD_3_OD,): δ = 7.74 (d, *J* = 9.2 Hz, 1H), 7.21 (dd, *J* = 9.2 Hz, 2 Hz, 1H), 6.97 (s, 1H), 6.76 (s, 1H), 6.57 (br s, 1H), 4.55 (t, *J* = 8 Hz, 2H), 3.33 (m, 2H), 3.27 (m, 2H), 1.97 (quint, *J* = 7.6 Hz, 2H), 1.76 (sext, *J* = 7.2 Hz, 4H), 1.56 (sext, *J* = 7.6 Hz, 2H), 1.20 (t, *J* = 7.4 Hz, 3H), 1.07 (t, *J* = 7.4 Hz, 3H), 1.02 (t, *J* = 7.4 Hz, 3H). ^13^C NMR (101 MHz, CD_3_OD): δ = 154.7 (C), 152.2 (C), 140.6 (C), 139.9 (C), 136.7 (C), 134.1 (C), 132.6 (C), 132.3 (CH), 107.5 (CH), 104.1 (CH), 93.7 (CH), 90.6 (CH), 50.2 (CH_2_), 46.0 (CH_2_), 44.6 (CH_2_), 31.5 (CH_2_), 22.9 (CH_2_), 21.5 (CH_2_), 21.0 (CH_2_), 14.2 (CH_3_), 11.9 (CH_3_), 11.4 (CH_3_). HRMS (ESI+) calculated for [C^+^]: 366.2652 (C_22_H_32_N_5_^+^) found: 366.2650.

Compound **6**. A solution of **4b** (143 mg, 0.32 mmol, 1 equiv.) in methanol (30 mL) was hydrogenated (30 bars) in the presence of Pd/C (5 wt. %5 mol, 34 mg) and HCl (12 M, 0.3 mL) for 16 h. Then, the mixture was stirred under air for another 24 h. Pd/C was removed via filtration through a Celite^®^ plug, which was rinsed multiple times with methanol and dichloromethane. After removal of the solvent under reduced pressure, the resulting solid was purified using an alumina oxide gel chromatography column (neutral, activity I, eluent dichloromethane/methanol, 95:5, solid deposition). The pink fraction was collected, concentrated under vacuum, and repassed on the same type of chromatography column to yield 35 mg of product (0.08 mmol, 27% yield).

R_f_: 0.55 (Al_2_O_3_, dichloromethane / methanol, 95:5). ^1^H NMR (400 MHz, CD_3_OD,): δ = 7.85 (d, *J*^3^ = 9.2 Hz, 1H, CH), 7.56 (s, 1H, CH), 7.30 (d, *J*^3^ = 9.3 Hz, 1H, CH), 7.06 (s, 1H, CH), 6.66 (s, 1H, CH), 4.60 (t, *J*^3^ = 7.7 Hz, 2H, CH_2_), 3.42 (t, *J*^3^ = 7.1 Hz, 2H, CH_2_), 3.24 (q, *J*^3^ = 7.1 Hz, 4H, CH_2_), 2.04 (sext, *J*^3^ = 7.8 Hz, 2H, CH_2_), 1.81 (sext, *J*^3^ = 7.1 Hz, 2H, CH_2_), 1.26 (t, *J*^3^ = 7.1 Hz, 3H, CH_3_), 1.12 (m, 9H, CH_3_). ^13^C NMR (101 MHz, CD_3_OD): δ = 157.2 (C), 156.8 (C), 143.1 (C), 137.9 (C), 137.5 (C), 136.3 (C), 134.7 (C), 134.3 (CH), 124.8 (2CH), 93.9 (2CH), 50.0 (CH_2_), 47.3 (CH_2_), 46.1 (CH_2_), 22.9 (CH_2_), 20.7 (CH_2_), 12.0 (CH_3_), 11.9 (CH_3_), 11.4 (CH_3_). HRMS (ESI+) calculated for [C^+^]: 366.2652 (C_22_H_32_N_5_^+^) found: 610.4482.

Compound **7**. A solution of **4c** (225 mg, 0.5 mmol, 1 equiv.) in methanol (40 mL) was hydrogenated (20 bars) in the presence of Pd/C (5 wt. %5 mol, 54 mg) and HCl (12 M, 0.4 mL) for 8 h. Then, the mixture was stirred under air for another 16 h. Pd/C was removed via filtration through a Celite^®^ plug, which was rinsed multiple times with methanol and dichloromethane. After removal of the solvent under reduced pressure, the resulting solid was purified using an alumina oxide gel chromatography column (neutral, activity I, eluent dichloromethane/methanol, 95:5, solid deposition). The purple fraction was collected, concentrated under vacuum, and precipitated in pentane, and the precipitate was collected via vacuum filtration to afford 173 mg of product (0.43 mmol, 86% yield).

R_f_: 0.4 (Al_2_O_3_, dichloromethane/methanol, 95:5). ^1^H NMR (400 MHz, CD_3_OD): δ = 7.90 (d, *J*^3^ = 9.5 Hz, 1H, CH), 7.48 (dd, *J*^3^ = 9.5 Hz, *J^4^* = 2.4 Hz, 1H, CH), 7.03 (s, 1H, CH), 6.86 (s, 1H, CH), 6.69 (d, *J^4^* = 2.4 Hz, 1H, CH), 4.66 (t, *J*^3^ = 7.8 Hz, 2H, CH_2_), 3.74 (q, *J*^3^= 7 Hz, 4H, CH_2_), 3.32 (m, 2H, CH_2_), 2.06 (sext, *J*^3^ = 7.9 Hz, 2H, CH_2_), 1.87 (sext, *J*^3^ = 7 Hz, 2H, CH_2_), 1.36 (t, *J*^3^ = 7.1 Hz, 6H, CH_3_), 1.25 (t, *J*^3^ = 7.1 Hz, 3H, CH_3_), 1.13 (t, *J*^3^ = 7.1 Hz, 3H, CH_3_). ^13^C NMR (101 MHz, CD_3_OD): δ = 152.6 (C), 152.5 (C), 140.7 (C), 140.4 (C), 136.1 (C), 133.6 (CH), 133.0 (C), 132.8 (C), 118.7 (CH), 104.2 (CH), 93.7 (CH), 92.2 (CH), 50.0 (CH_2_), 46.7 (CH_2_), 46.4 (CH_2_), 22.5 (CH_2_), 21.2 (CH_2_), 12.8 (CH_3_), 12.1 (CH_3_), 11.4 (CH_3_). HRMS (ESI+) calculated for [C^+^]: 366.2652 (C_22_H_32_N_5_^+^) found: 366.2654.

## Figures and Tables

**Table 1:** Optical properties of dyes **5**–**7** recorded in acetonitrile, dimethylformamide, and water.

Dye	Solvent	λ_max_ (ε, M^−1^ cm^−1^)	λ_em_	Φ ^[a]^	Stokes Shift (cm^−1^)
**5**	MeCN DMF H_2_O	552 (33,740) 550 (−) 566 (−)	644 639 668	0.58 0.61 −	2590 2530 2700
**6**	MeCN DMF H_2_O	531 (47,960) 545 (−) 538 (−)	642 622 660	0.006 0.006 −	3250 2270 3440
**7**	MeCN DMF H_2_O	579 (38,790) 572 (−) 583 (−)	657 651 697	0.68 0.67 −	2050 2120 2800

^[a]^ Relative fluorescence quantum yields measured with rhodamine B as reference (Φ = 70% in MeOH, λ_ex_ = 530 nm).

## Data Availability

Data are contained within the article and Supplementary Materials.

## Supplementary Materials

The following supporting information can be downloaded at https://www.mdpi.com/article/10.3390/molecules29204830/s1. Figures S1–S25: NMR spectra for the isolated precursors and final compounds; Figures S26–S32: HRMS spectra for the isolated precursors and final compounds; Table S1: Crystal data and structure refinement for **6**; Table S2: Crystal data and structure refinement for **7**; Figure S33: Molecular packing motif for compound **6**; Figure S34: Asymmetric crystallization unit of **7** composed of two independent molecules; Figure S35: Molecular packing motif for compound **7**; Figures S36–S38: Normalized electronic absorption and emission spectra of **5**–**7** in DMF and H_2_O; Figures S39–S41: Fluorescence spectra of **5**–**7** in DMSO under gradual heating from 25 °C to 80 °C; Figure S42: Electronic absorption and emission spectra of **6** in acetonitrile with the gradual addition of HCl; Scheme S1: Structure of the emissive cationic and poorly-emissive neutral form of **6.** Crystal data for compounds **6** and **7** have been deposited in the CCDC database under deposition numbers 2380658 and 2380659, respectively. The cif files for **6** and **7** are available as Supplementary Information on request made to the authors.
