# Supplementary material for: Emerging Thermosensitive Probes Based on Triamino-Phenazinium Dyes
Source: Molecules. 2024 Oct 12;29(20):4830. doi: 10.3390/molecules29204830 (PMC11509897; doi:10.3390/molecules29204830)
Supplement: Supplementary file 1 [file molecules-29-04830-s001.zip › molecules-3207325-supplementary.pdf]

Supporting information

Emerging thermosensitive probes based on triamino-phenazinium dyes

Tatiana Munteanu [1], Frederic Brunel [1], Michel Camplo [1] and Olivier Siri [1,*]

[1] Aix Marseille Univ., CNRS UMR 7325 Centre Interdisciplinaire de Nanoscience de Marseille (CINaM), Campus de Luminy, 13288 Marseille cedex 09, France ; tatiana.munteanu@univ-amu.fr (T.M), frederic.brunel@univ-amu.fr (F.B.) ; michel.camplo@univ-amu.fr (M.C)

* Correspondence: olivier.siri@univ-amu.fr;

TABLE OF CONTENT

I. NMR SPECTRA 2

II. MASS SPECTROMETRY 16

III. X-RAY DIFFRACTION 20

IV. ADDITIONAL OPTICAL SPECTRA 23

I. NMR SPECTRA

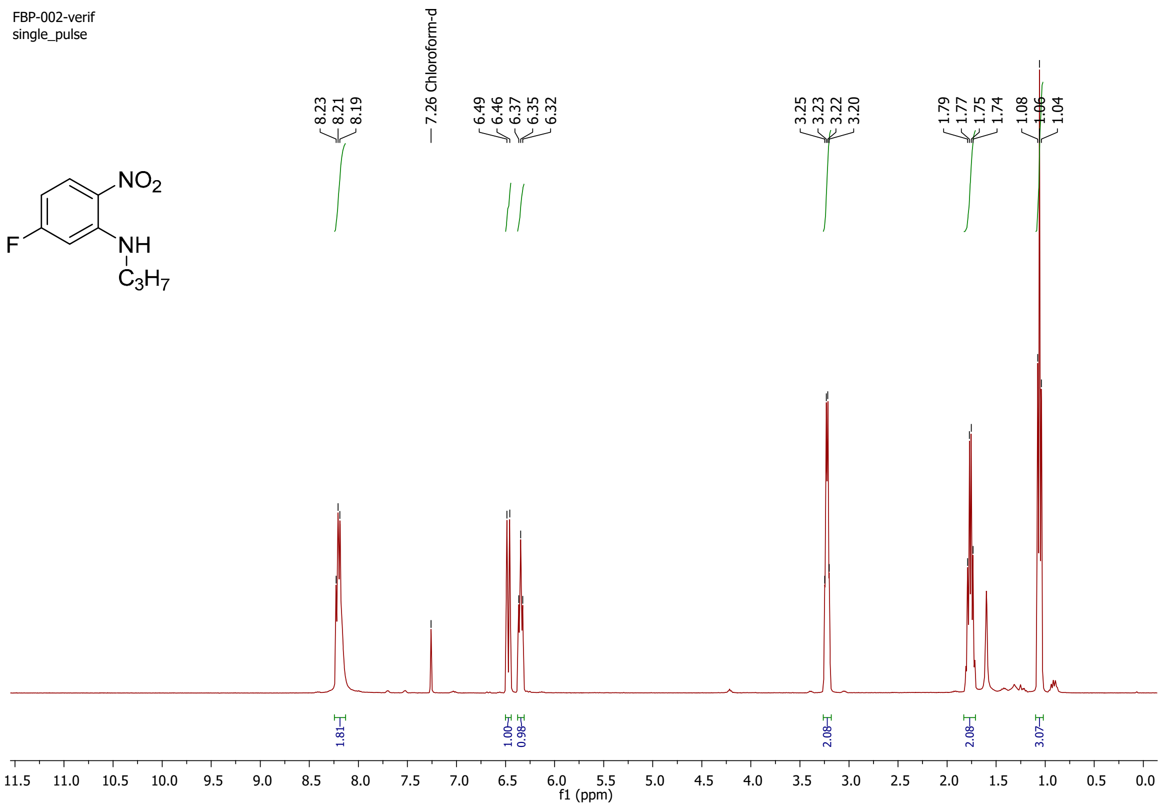

Figure S 1. ^{1}H NMR (400 MHz, $CDCl_3$) of **1**

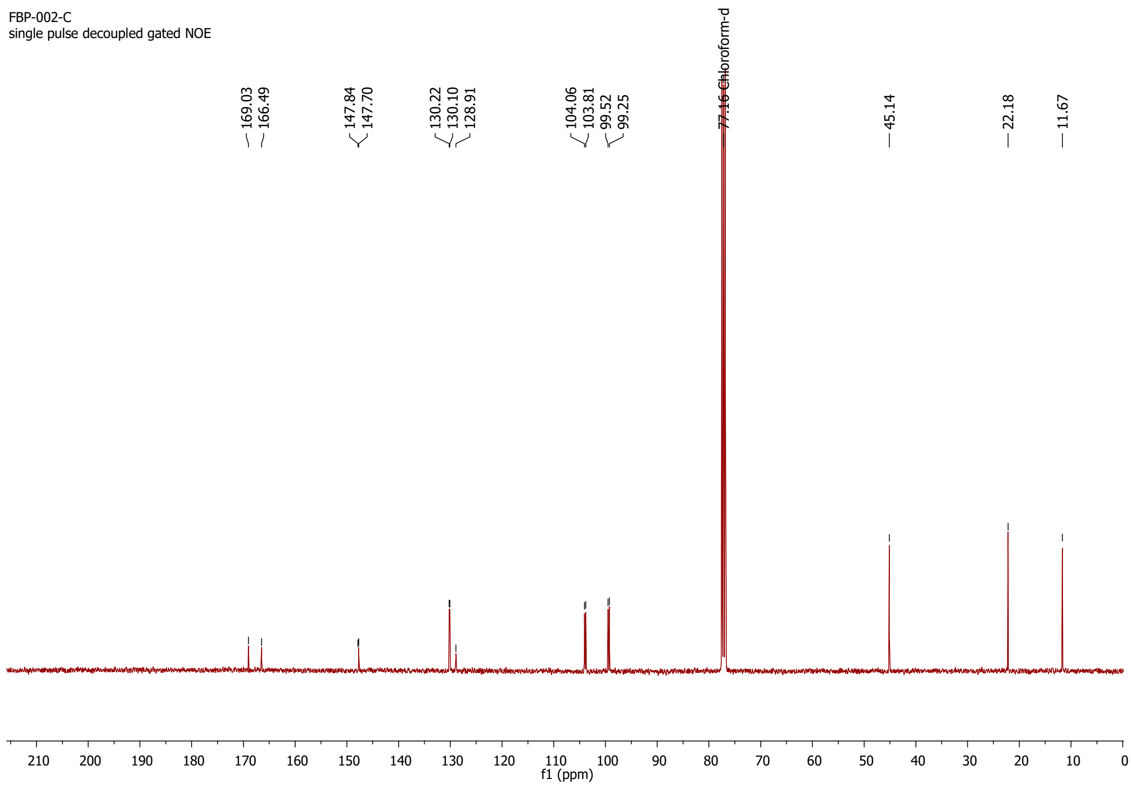

Figure S 2. ^{13}C NMR (101 MHz, $CDCl_3$) of **1**

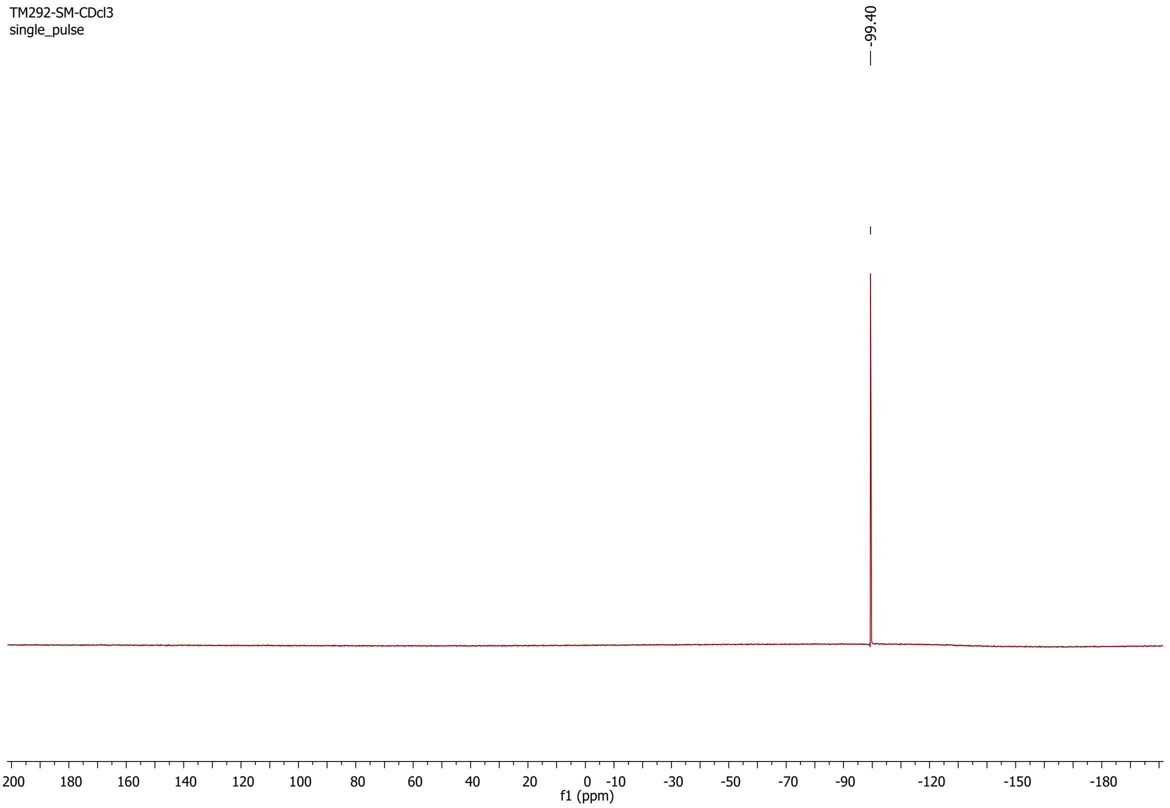

Figure S 3 ^{19}F NMR (376 MHz, $CDCl_3$) of **1**

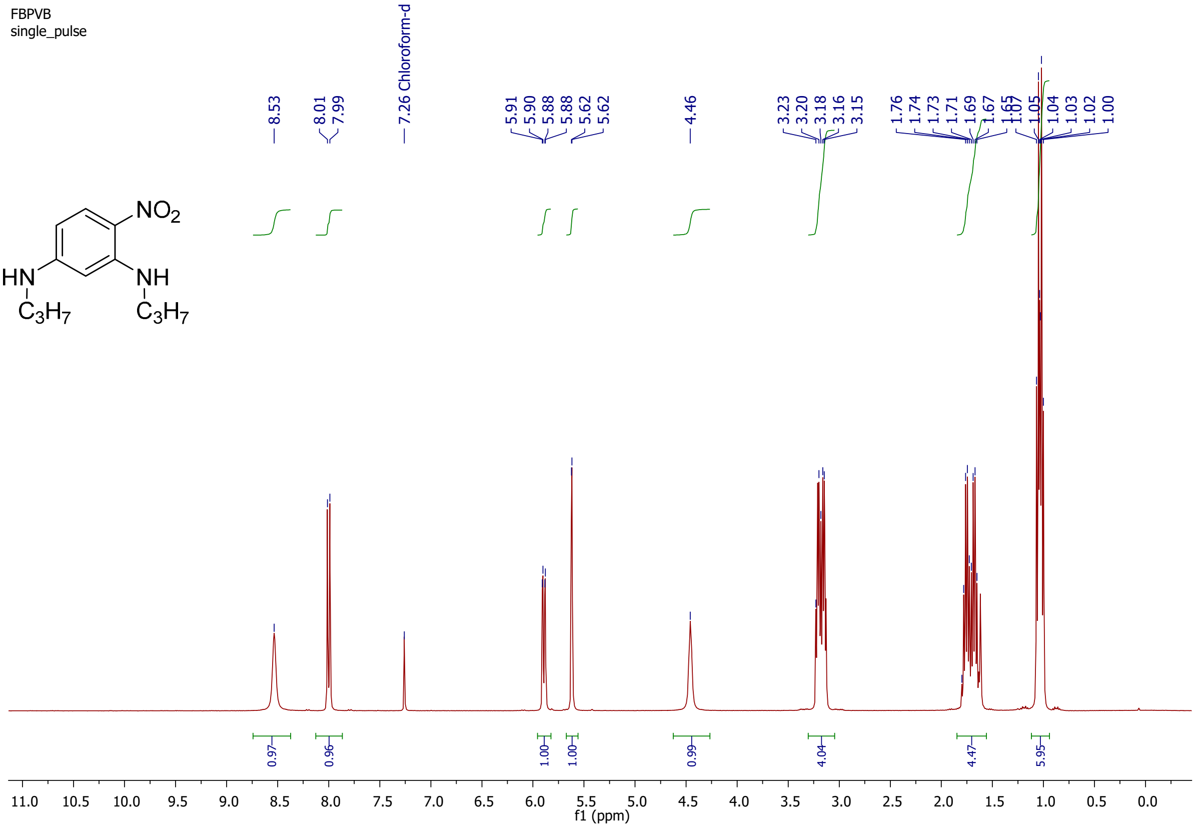

Figure S 4 ^{1}H NMR (400 MHz, $CDCl_3$) of **2a**

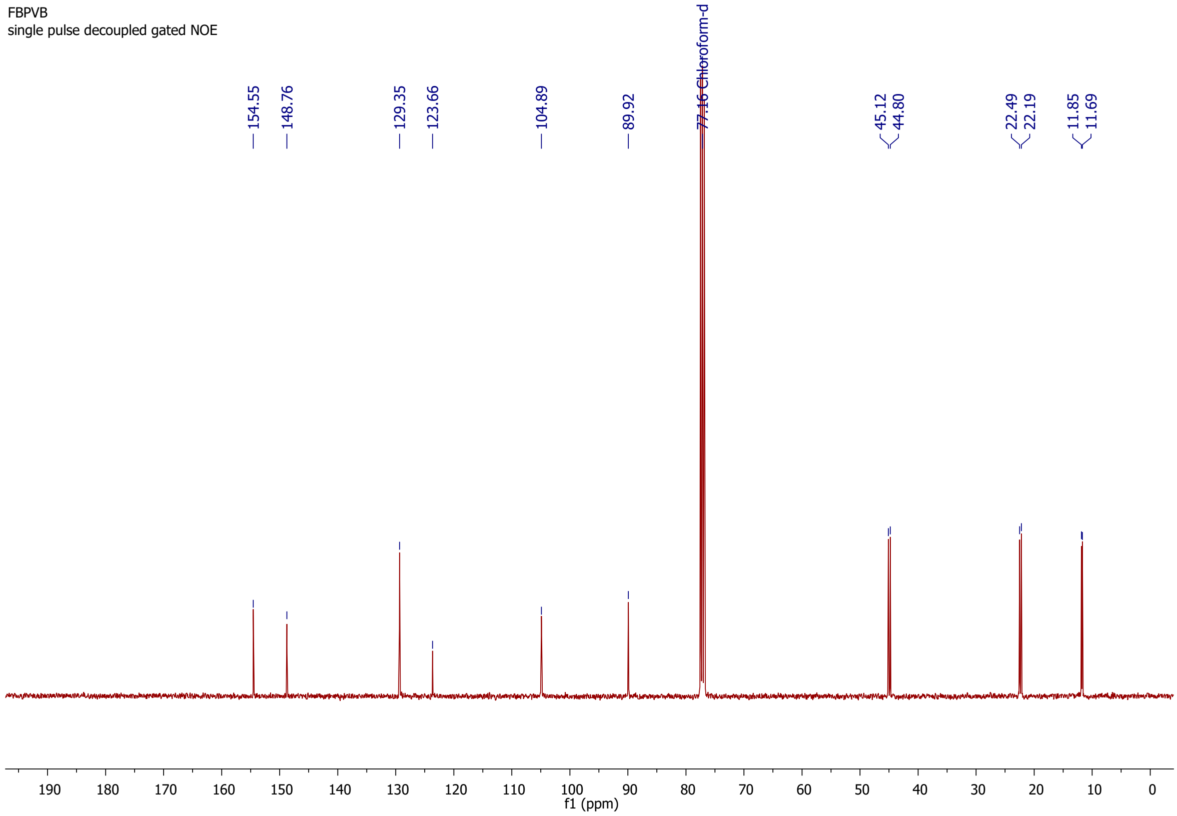

Figure S 5 ^{13}C NMR (101 MHz, $CDCl_3$) of **2a**

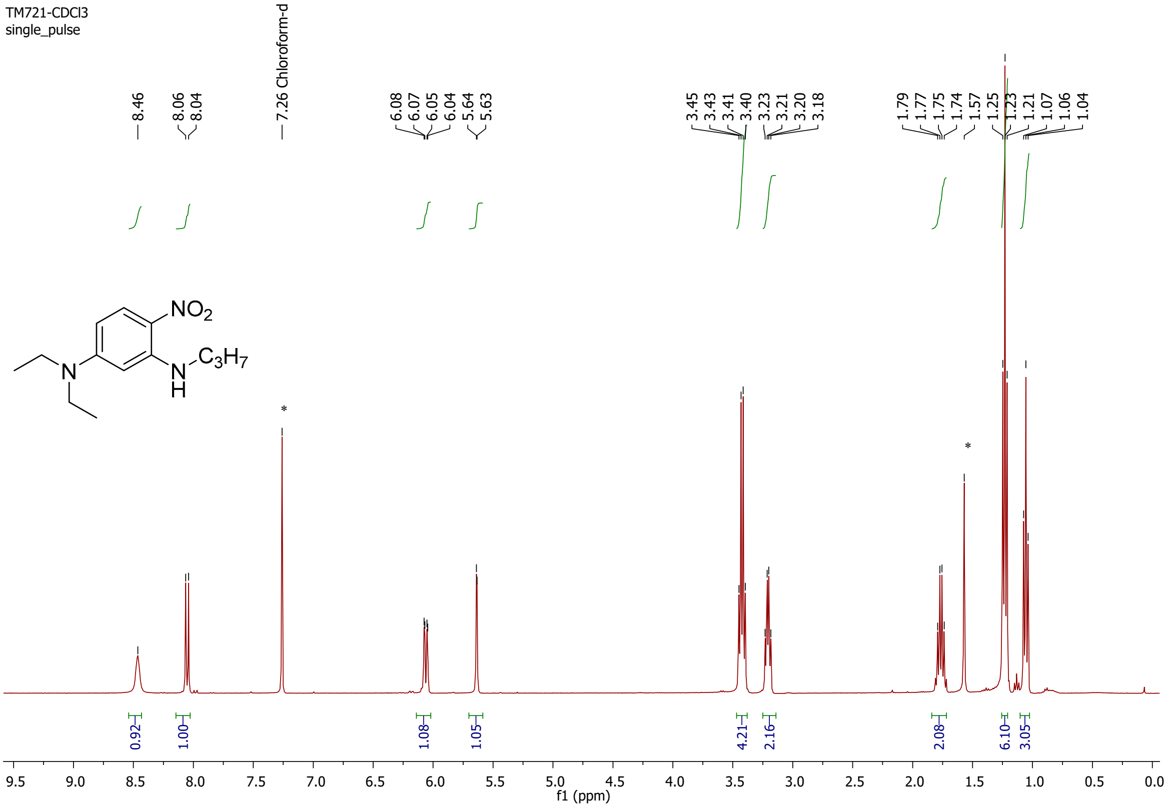

Figure S 6 **^{1}H NMR** (400 MHz, $CDCl_3$) of **2b**

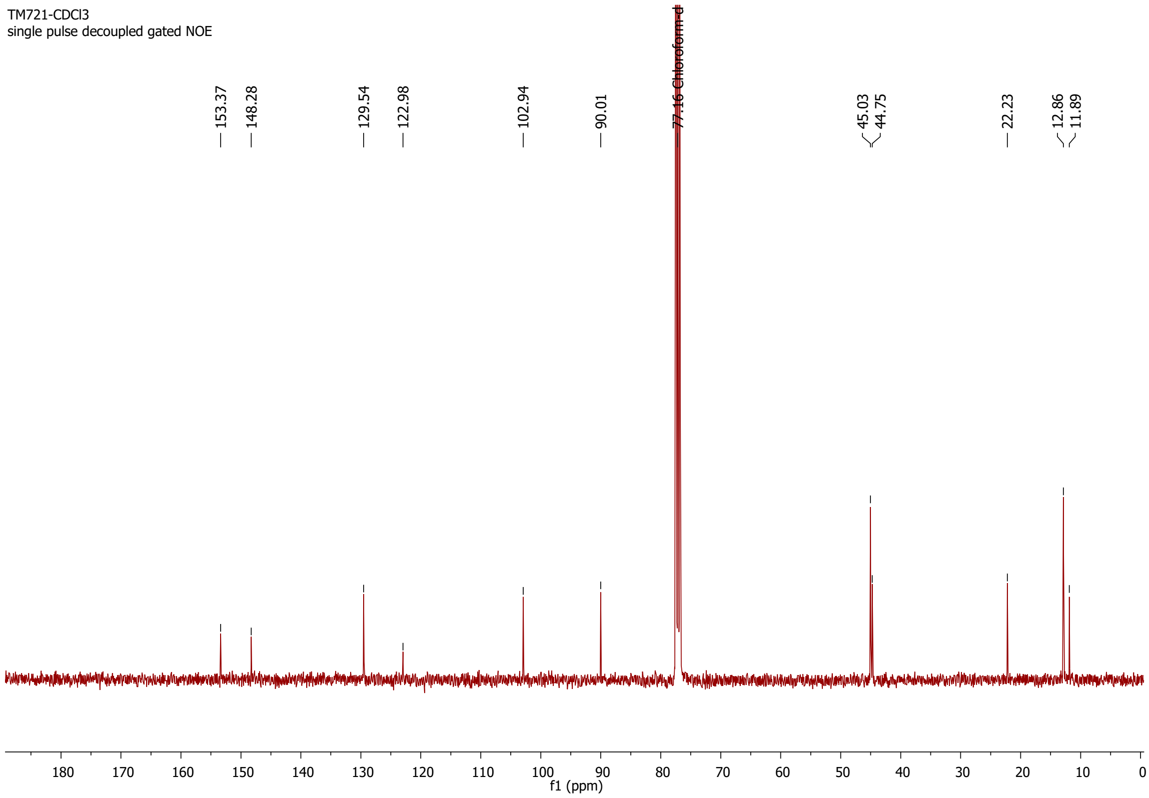

Figure S 7 **^{13}C NMR** (101 MHz, $CDCl_3$) of **2b**

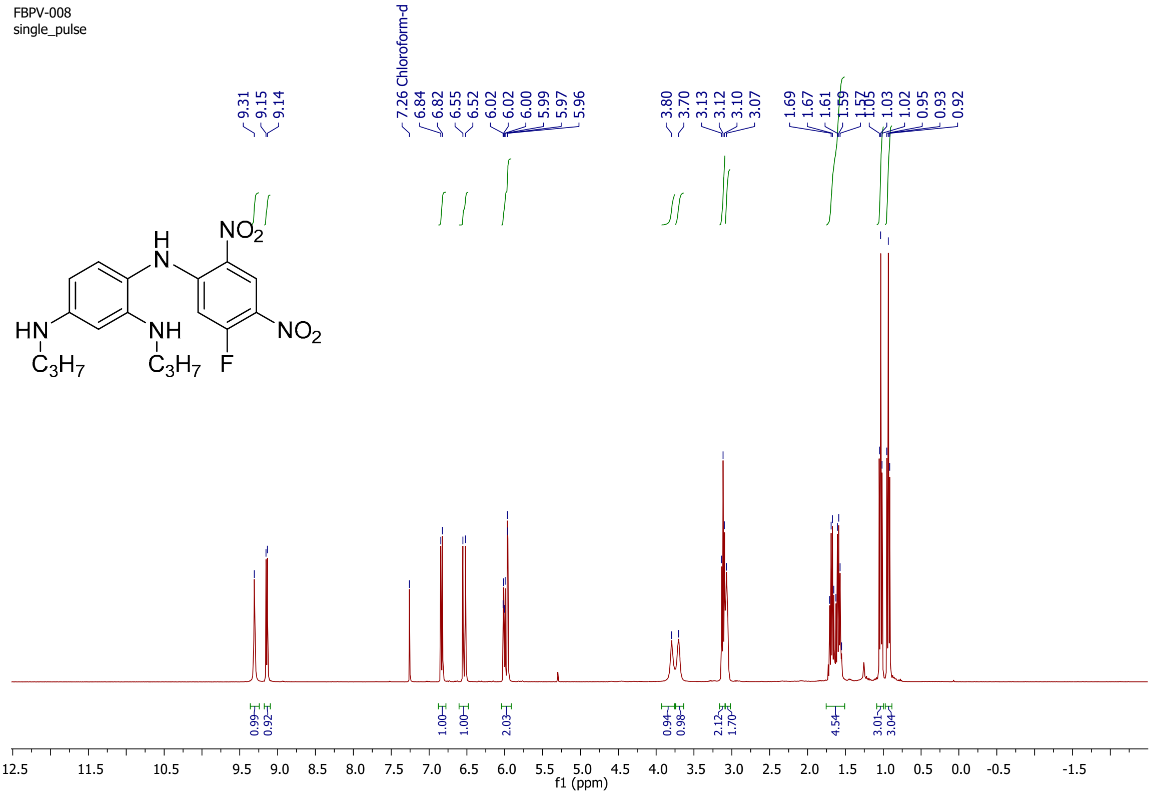

Figure S 8 ^{1}H NMR (400 MHz, $CDCl_3$) of **3a**

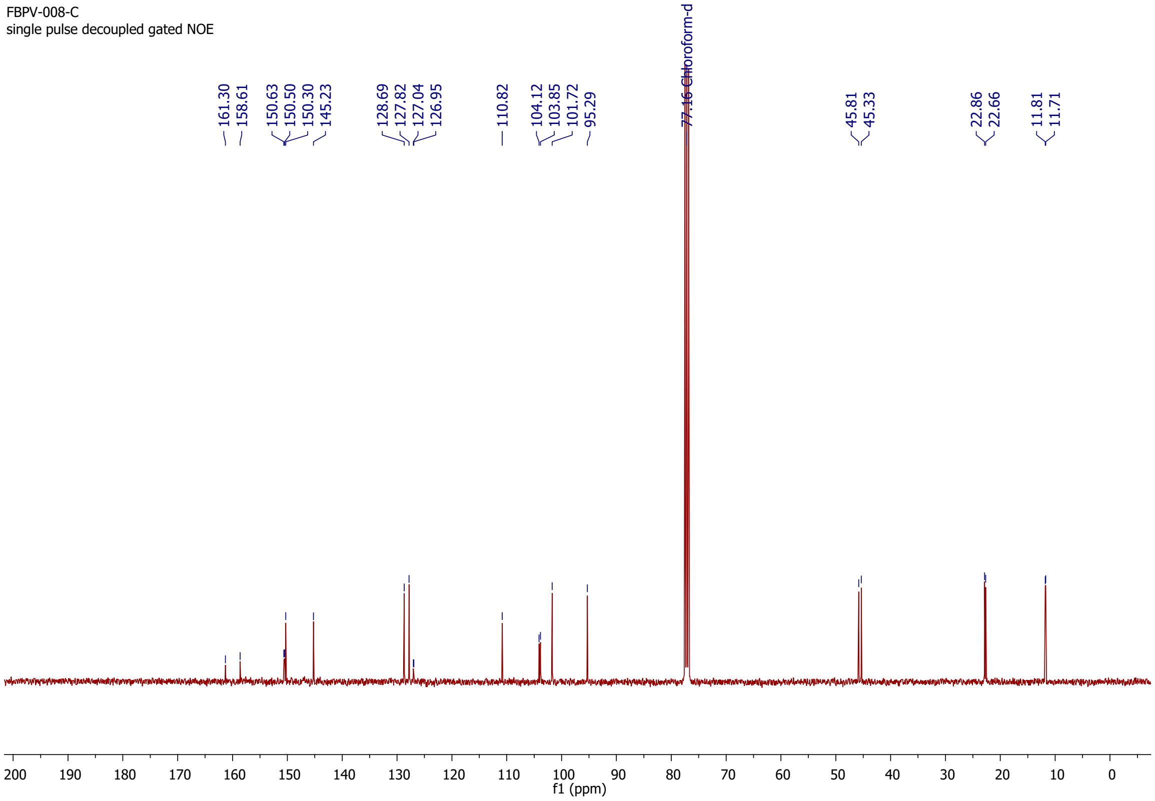

Figure S 9 ^{13}C NMR (101 MHz, $CDCl_3$) of **3a**

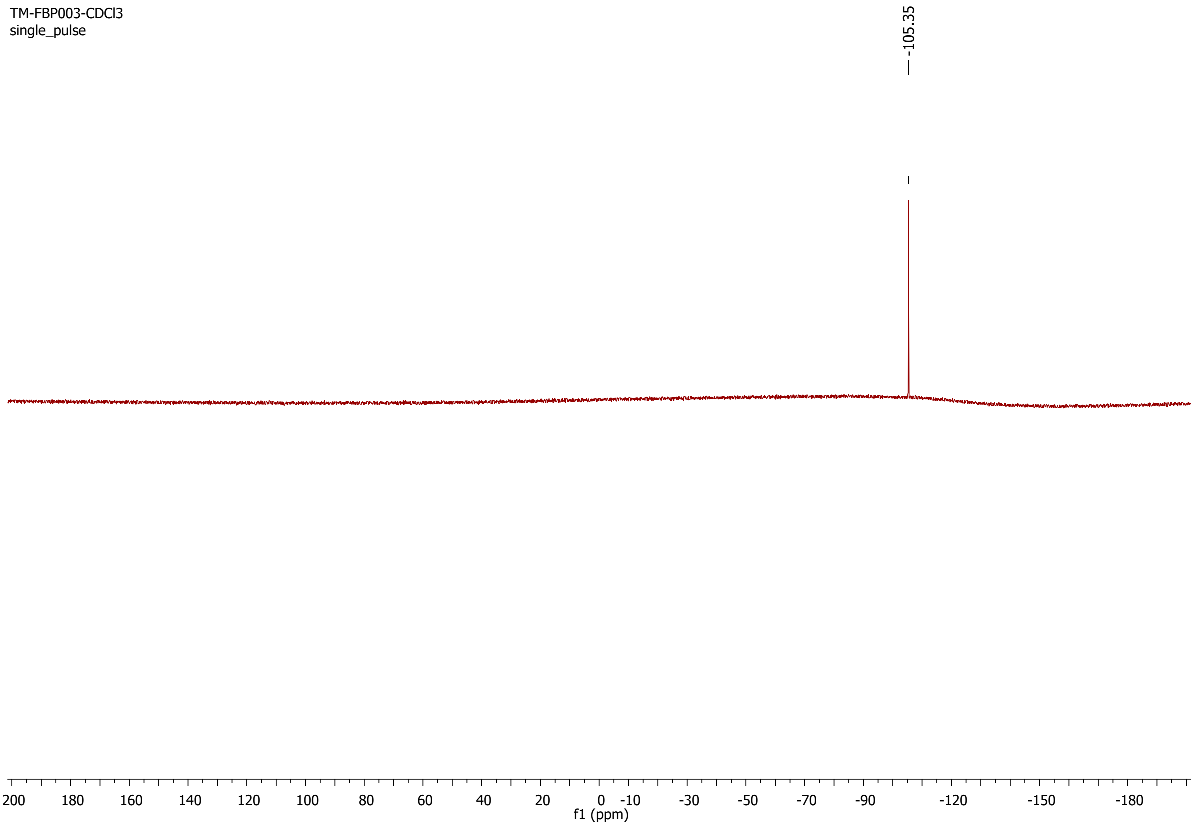

Figure S 10 ^{19}F NMR (376 MHz, $CDCl_3$) of **3a**

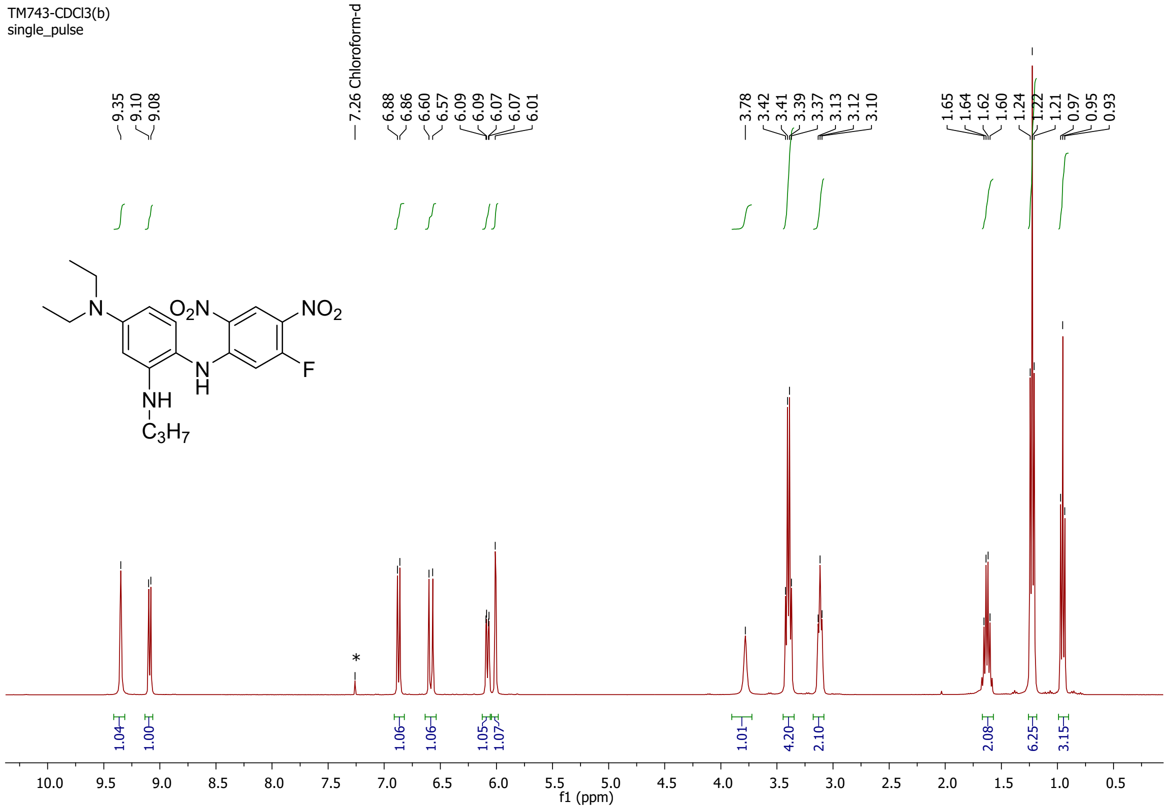

Figure S 11 **^{1}H NMR** (400 MHz, $CDCl_3$) of **3b**

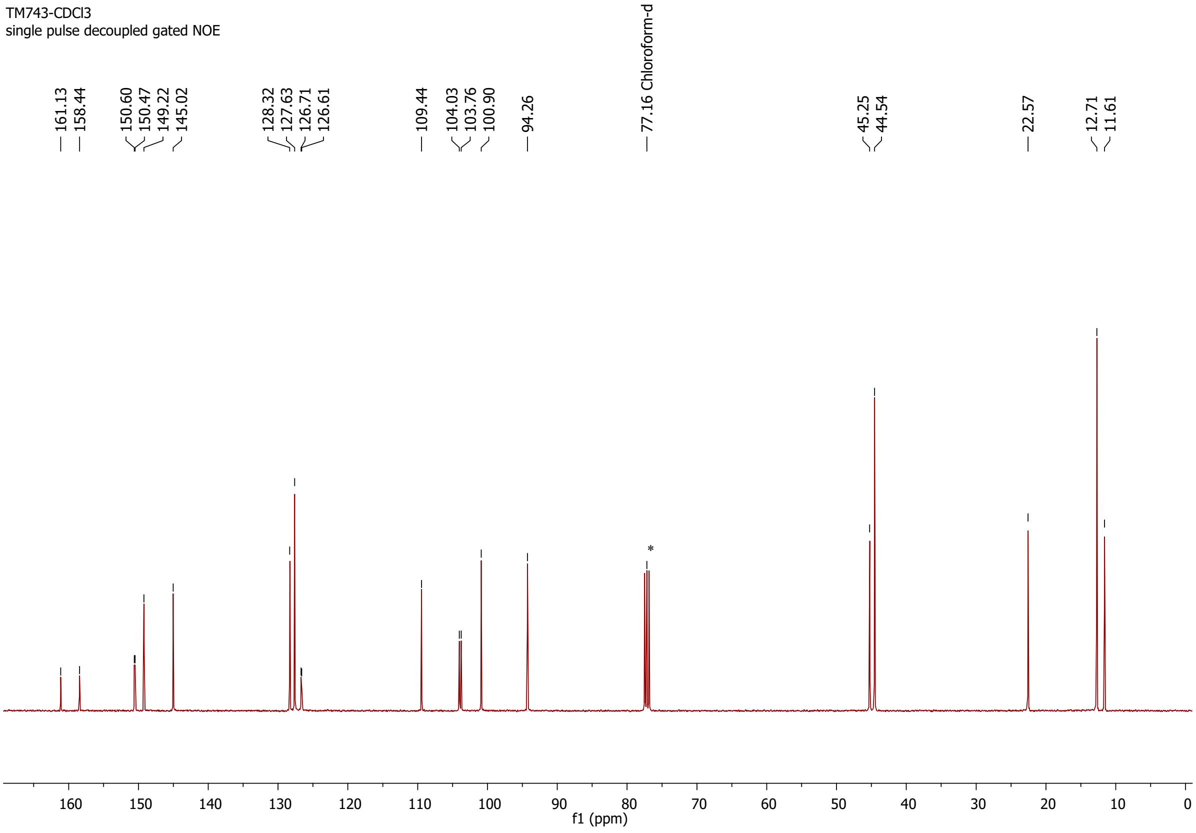

Figure S 12 **^{13}C NMR** (101 MHz, $CDCl_3$) of **3b**

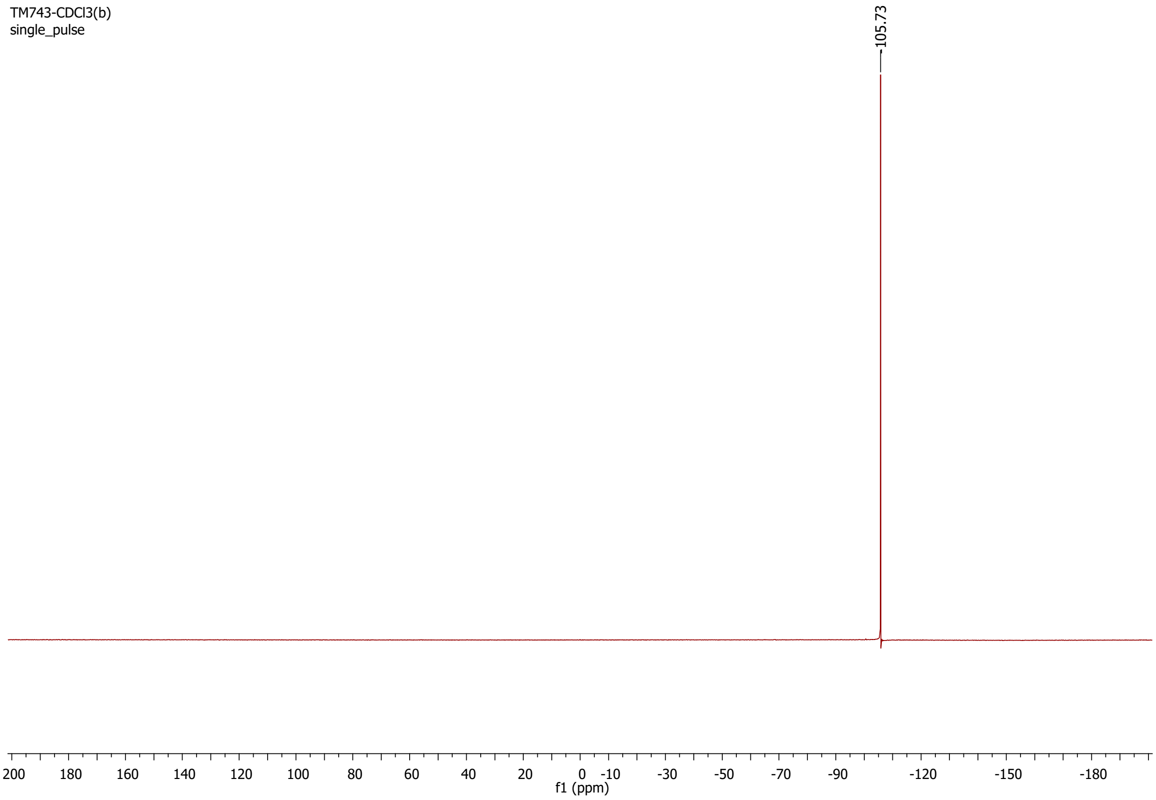

Figure S 13 ^{19}**F NMR** (376 MHz, $CDCl_3$) of **3b**

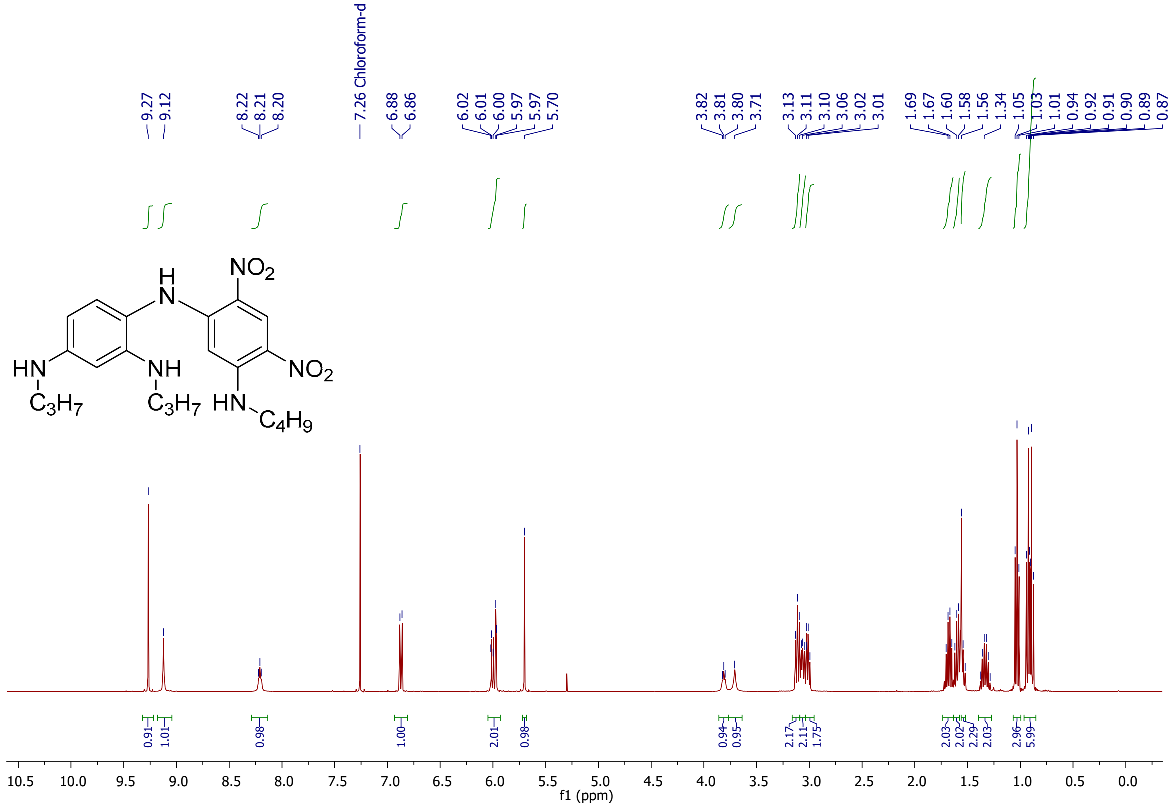

Figure S 14 ^{1}H NMR (400 MHz, $CDCl_3$) of **4a**

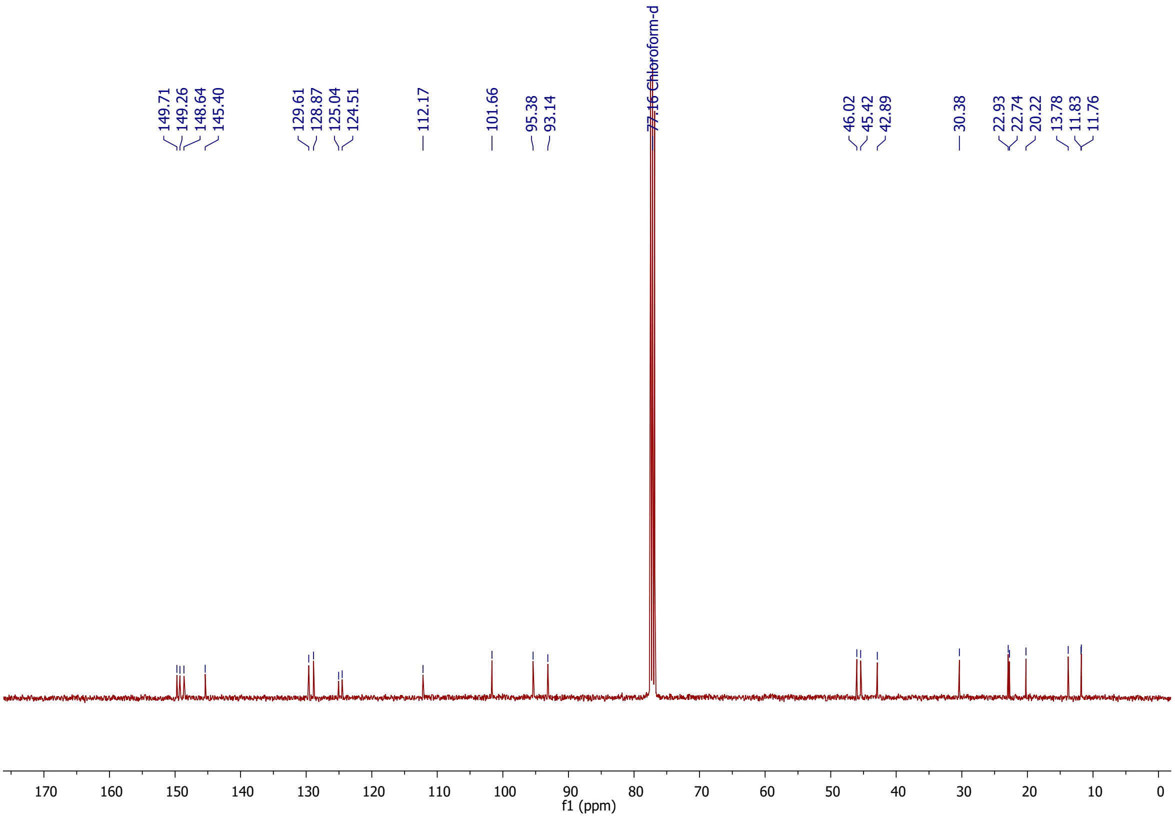

Figure S 15 ^{13}C NMR (101 MHz, $CDCl_3$) of **4a**

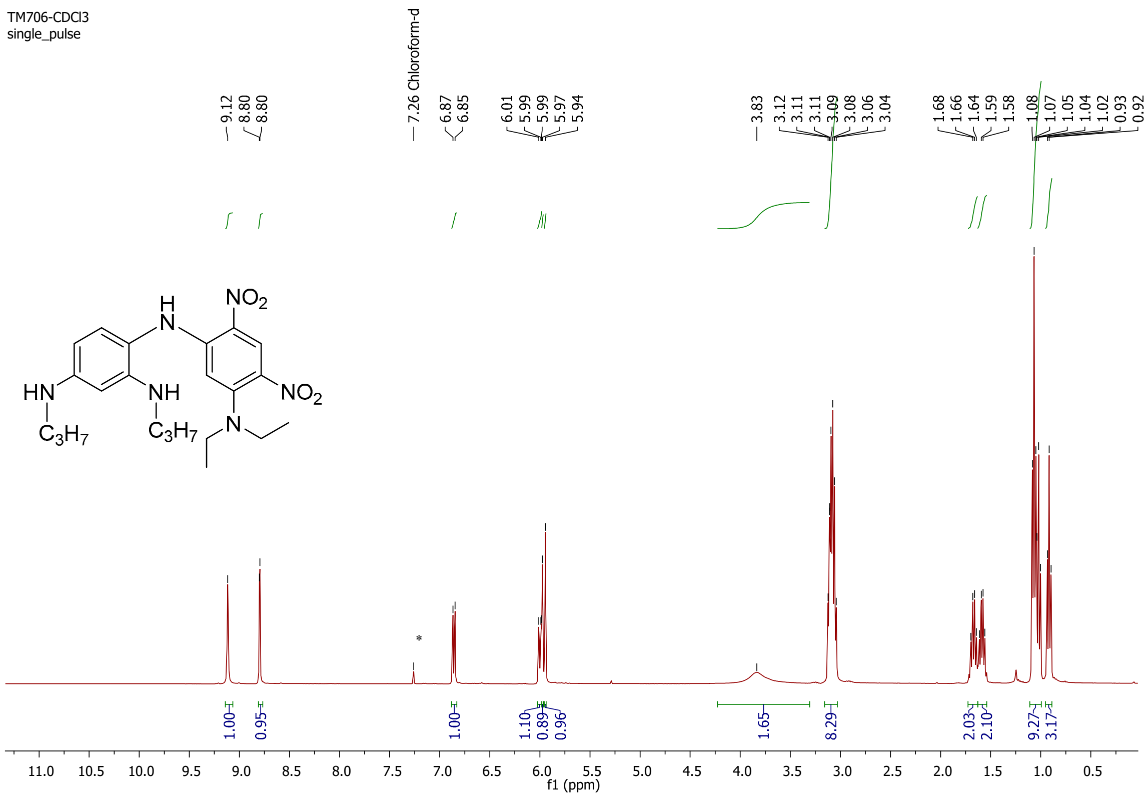

Figure S 16 ^{1}**H NMR** (400 MHz, $CDCl_3$) of **4b**

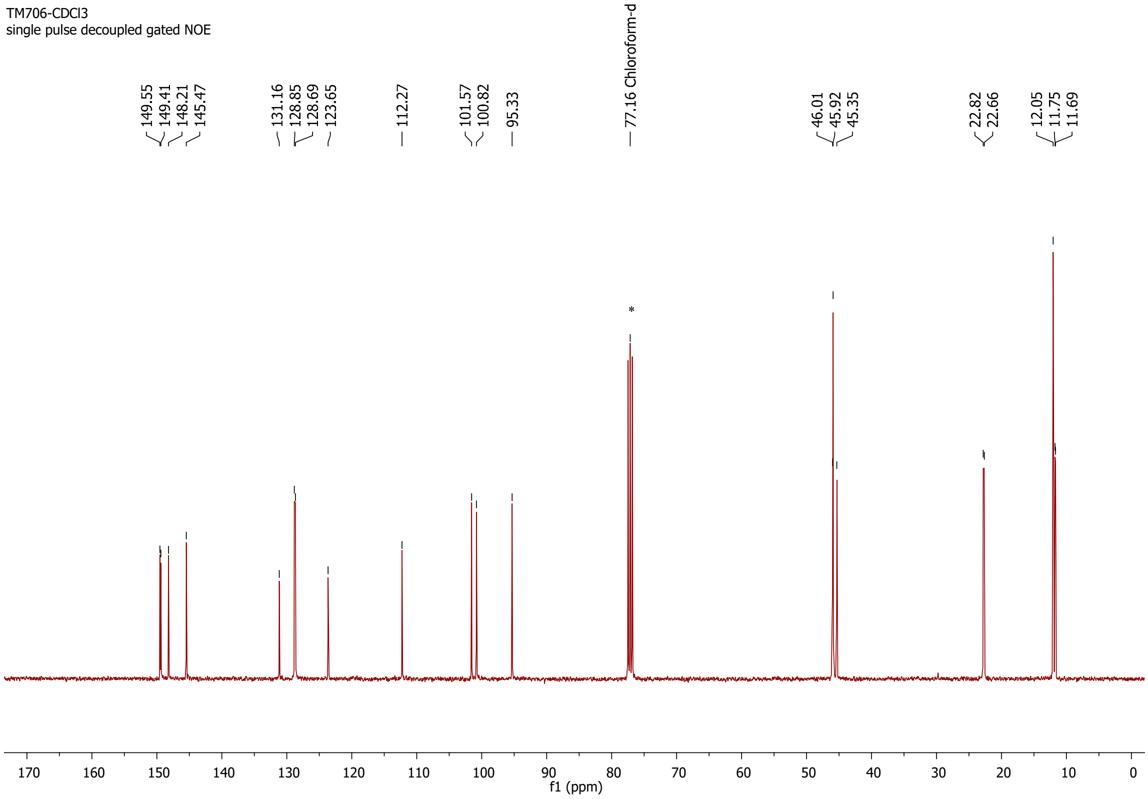

Figure S 17 ^{13}**C NMR** (101 MHz, $CDCl_3$) of **4b**

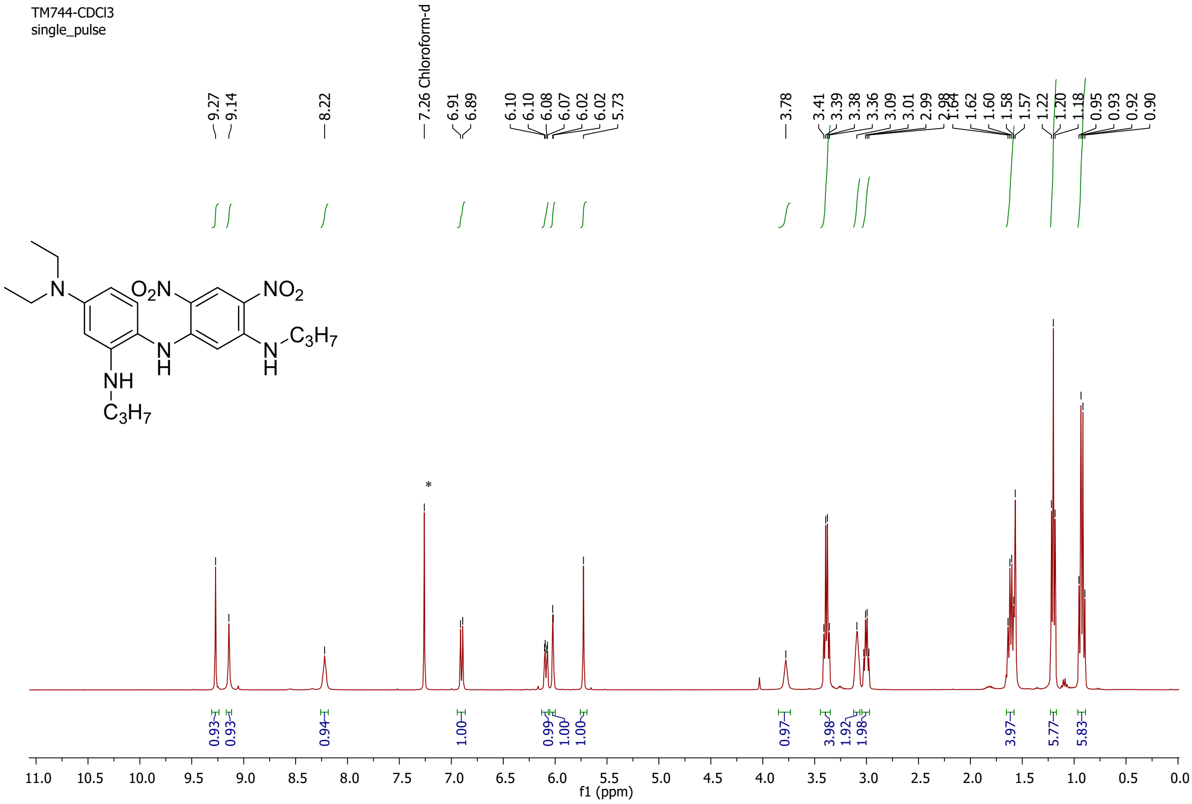

Figure S 18 **^{1}H NMR** (400 MHz, $CDCl_3$) of **4c**

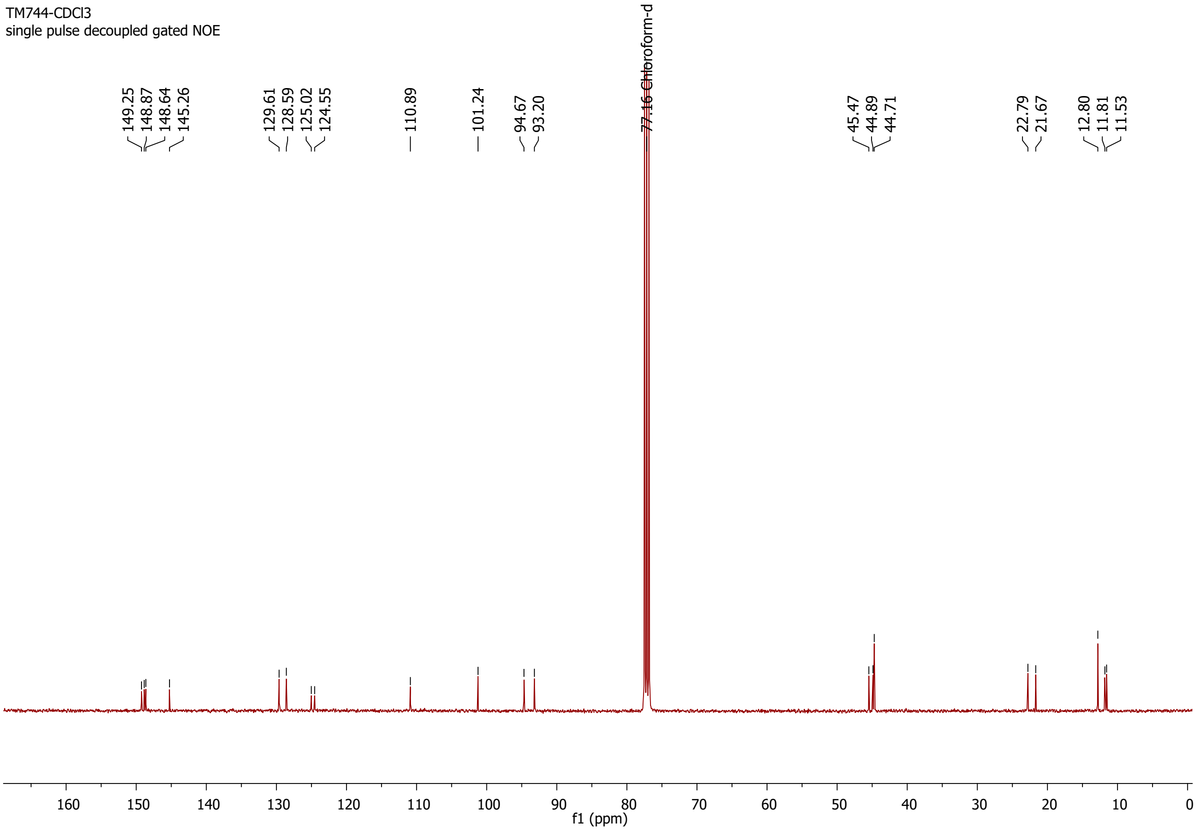

Figure S 19 **^{13}C NMR** (101 MHz, $CDCl_3$) of **4c**

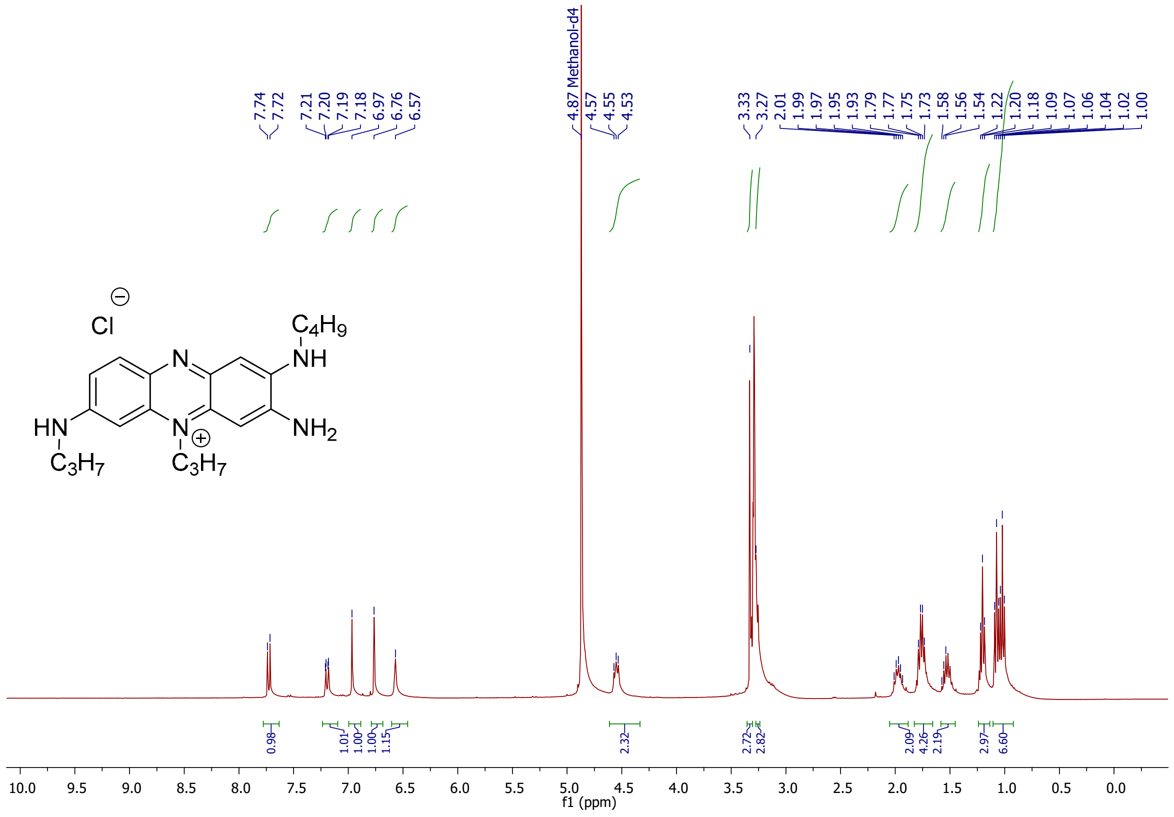

Figure S 20 ^{1}H NMR (400 MHz, CD_3OD) of **5**

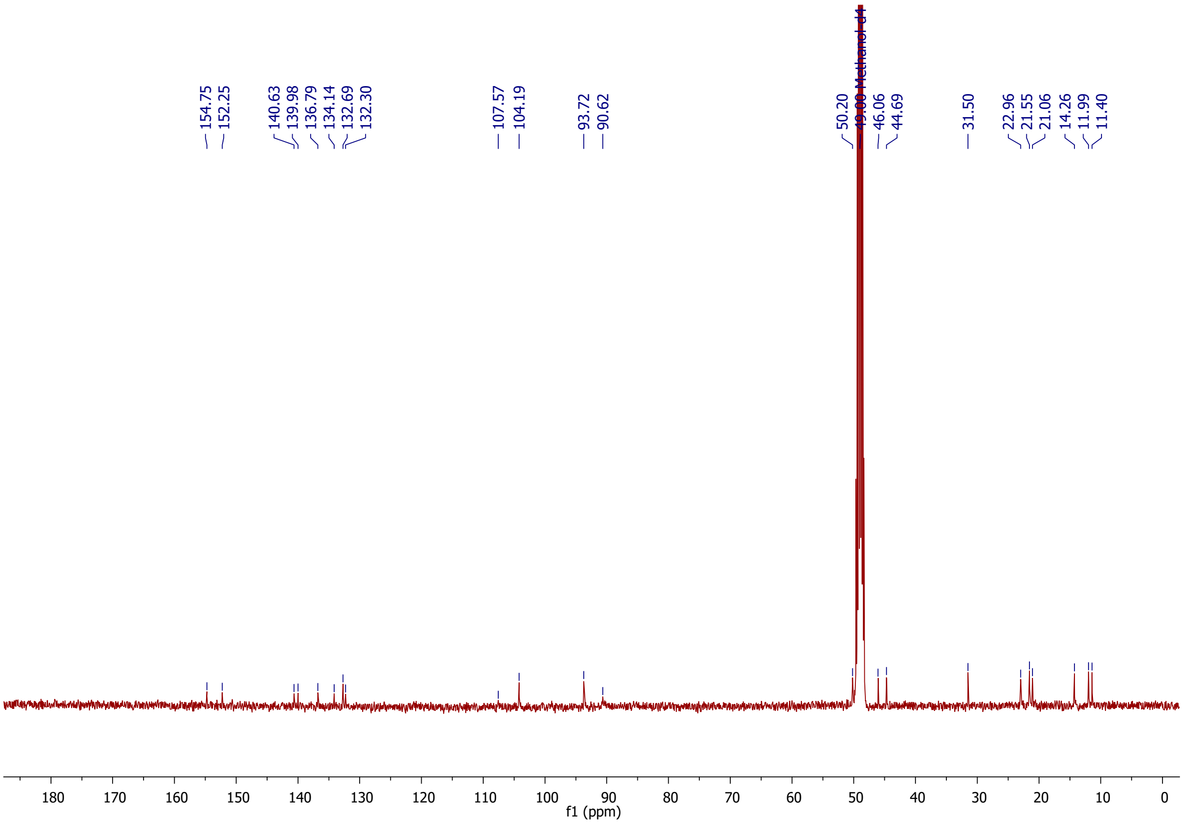

Figure S 21 ^{13}C NMR (101 MHz, CD_3OD) of **5**

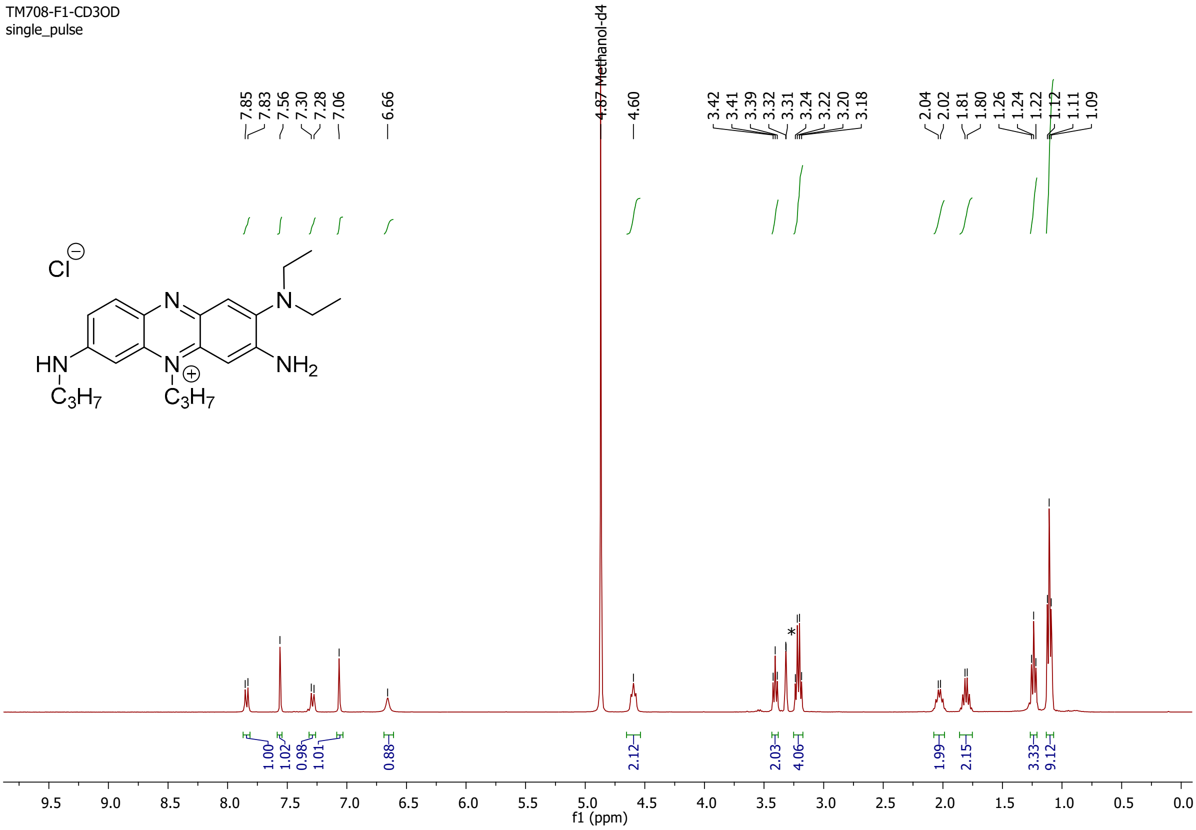

Figure S 22 **^{1}H NMR** (400 MHz, CD_3OD) of **6**

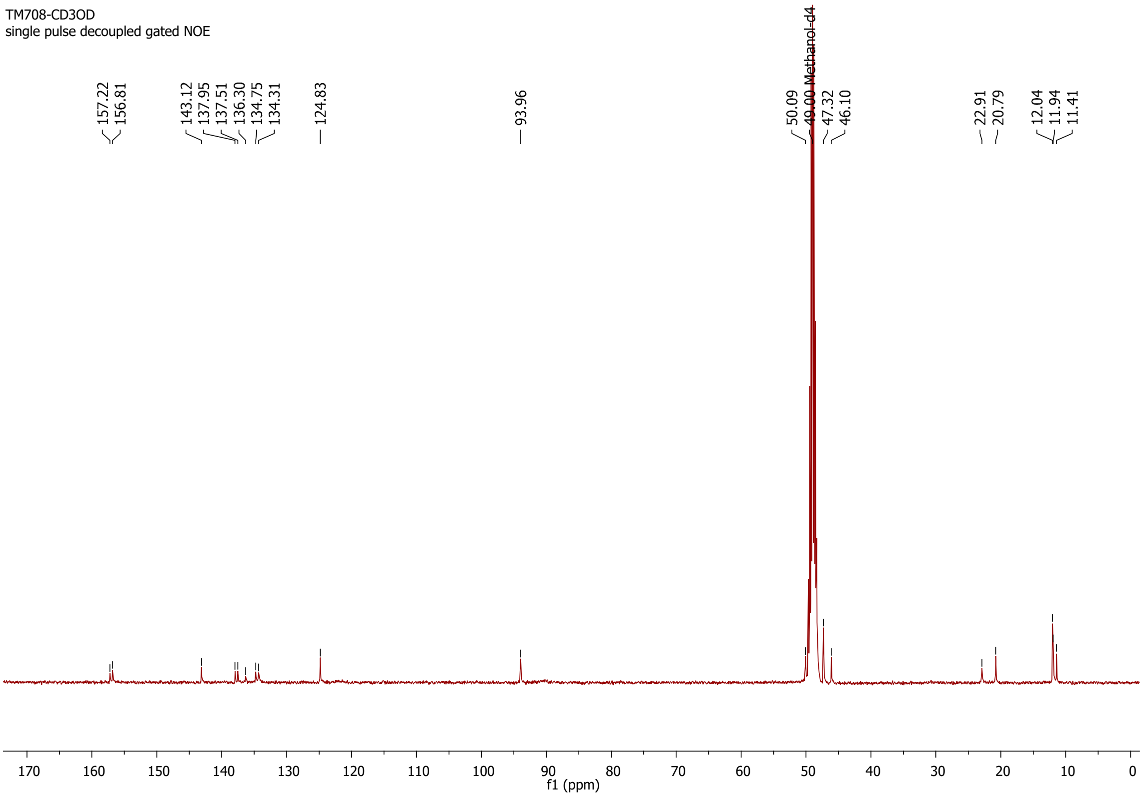

Figure S 23 **^{13}C NMR** (101 MHz, CD_3OD) of **6**

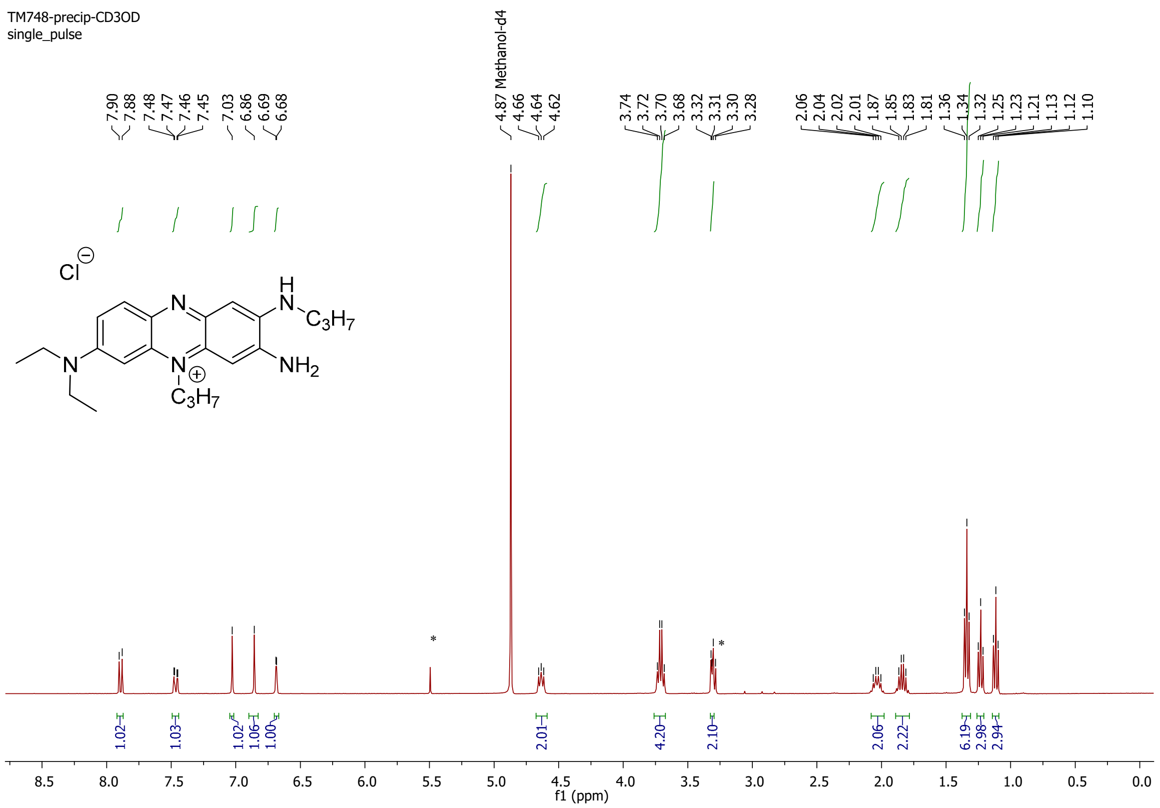

Figure S 24 ^{1}**H NMR** (400 MHz, CD_3OD) of **7**

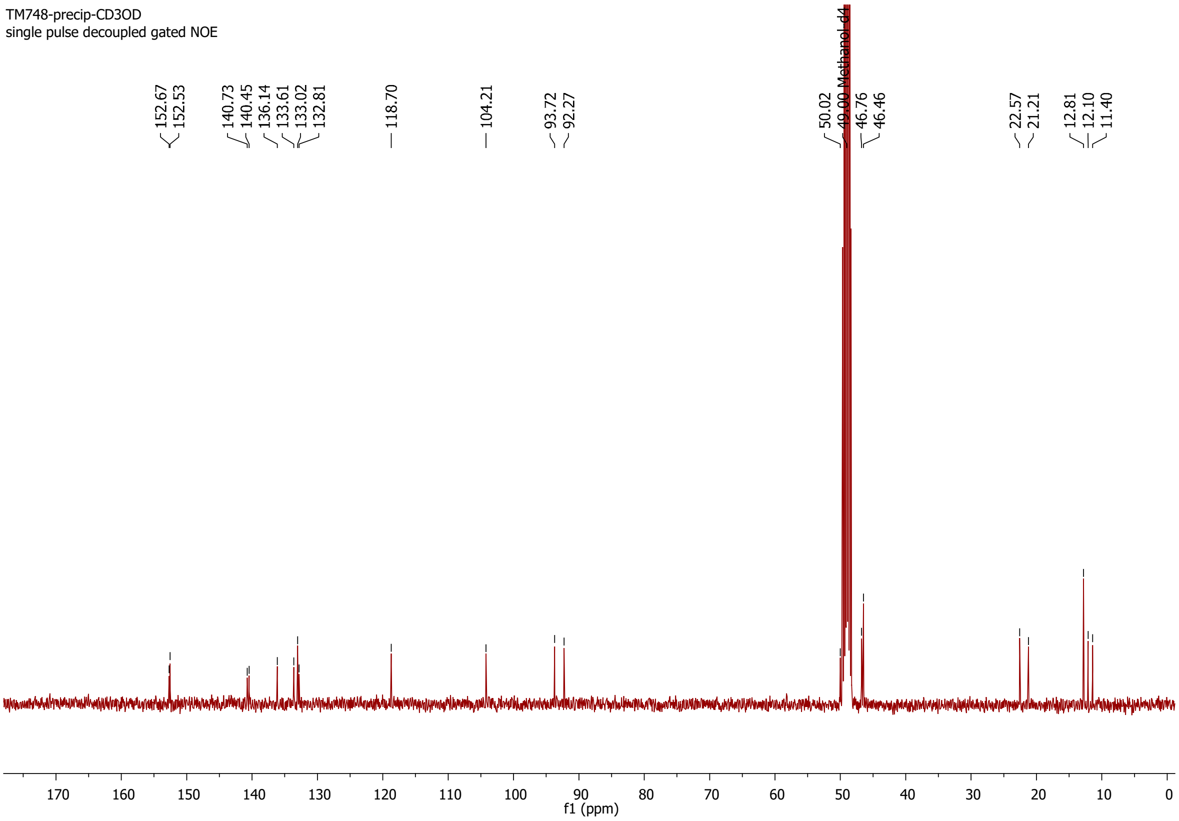

Figure S 25 ^{13}**C NMR** (101 MHz, CD_3OD) of **7**

II. MASS SPECTROMETRY

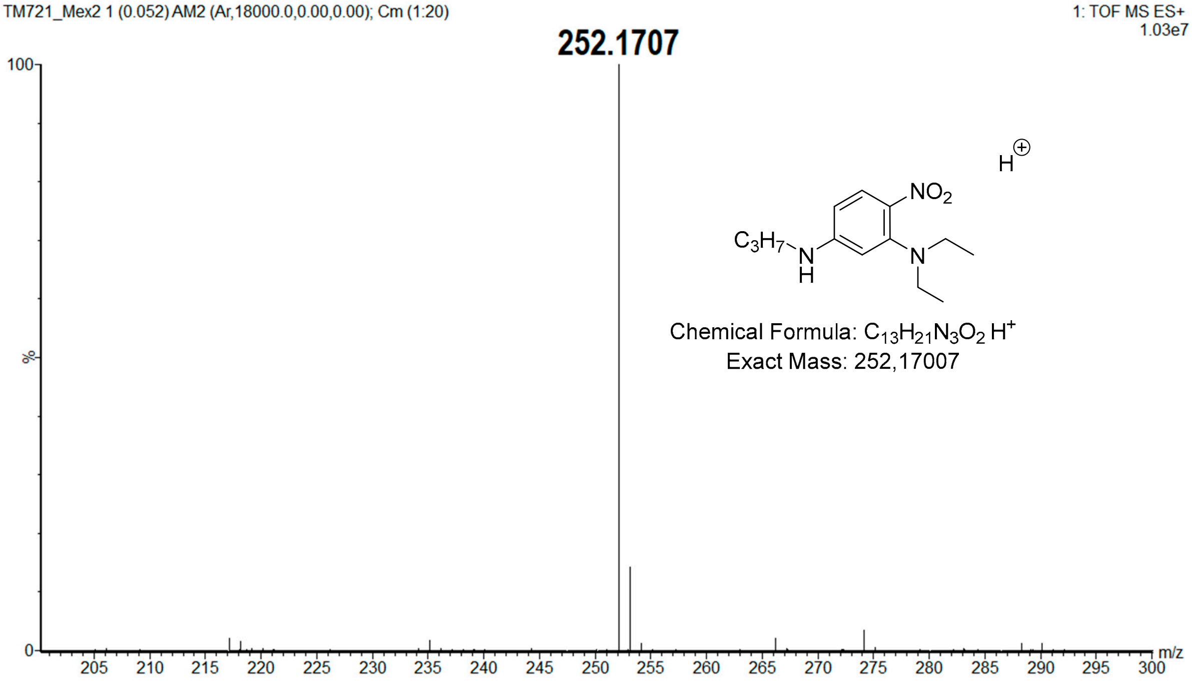

Figure S 26 HRMS spectrum of **2b**

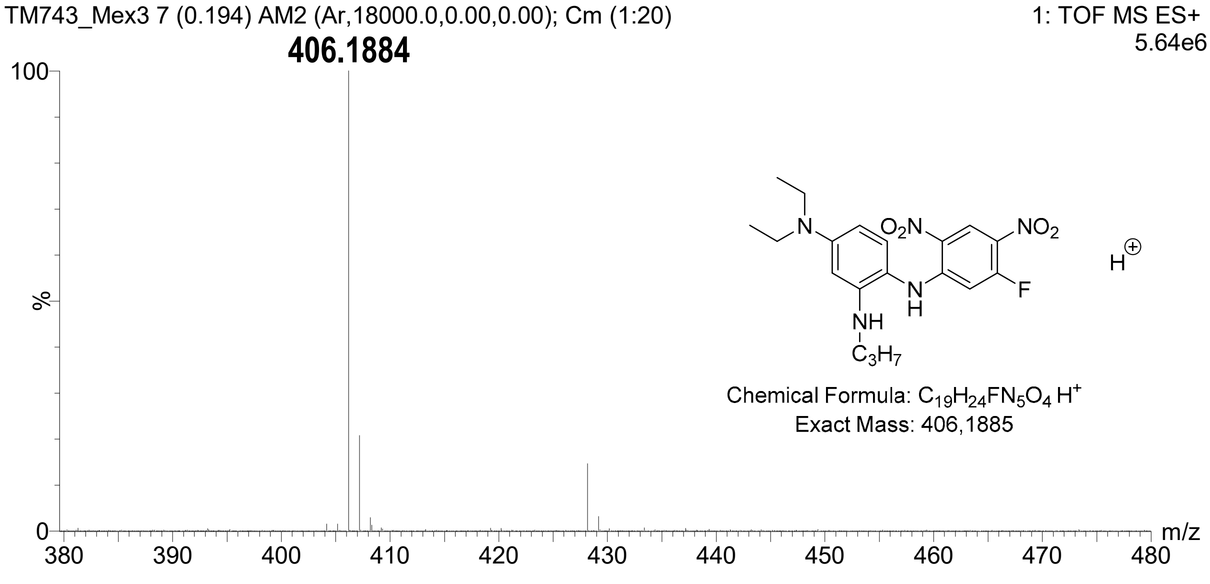

Figure S 27 HRMS spectrum of **3b**

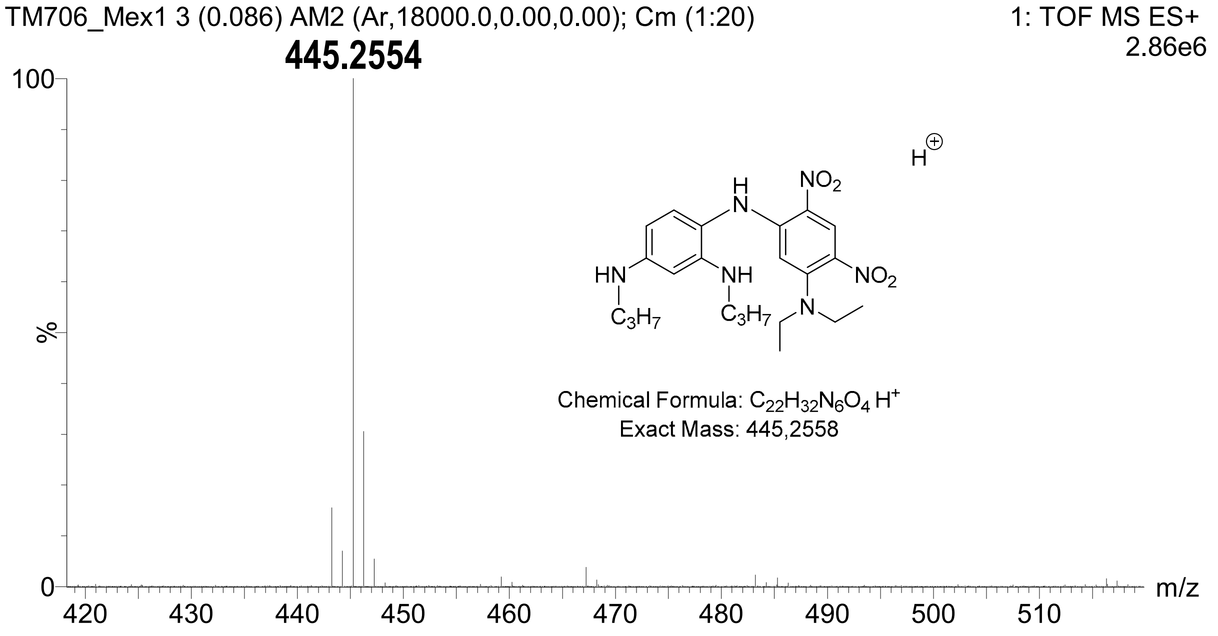

Figure S 28 HRMS spectrum of **4b**

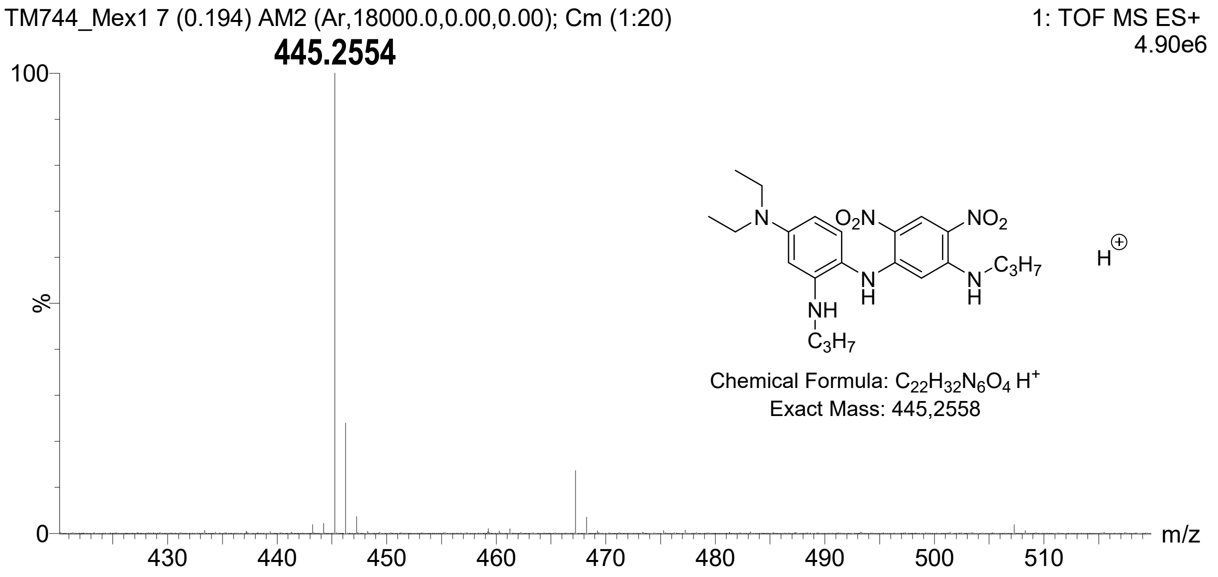

Figure S 29 HRMS spectrum of **4c**

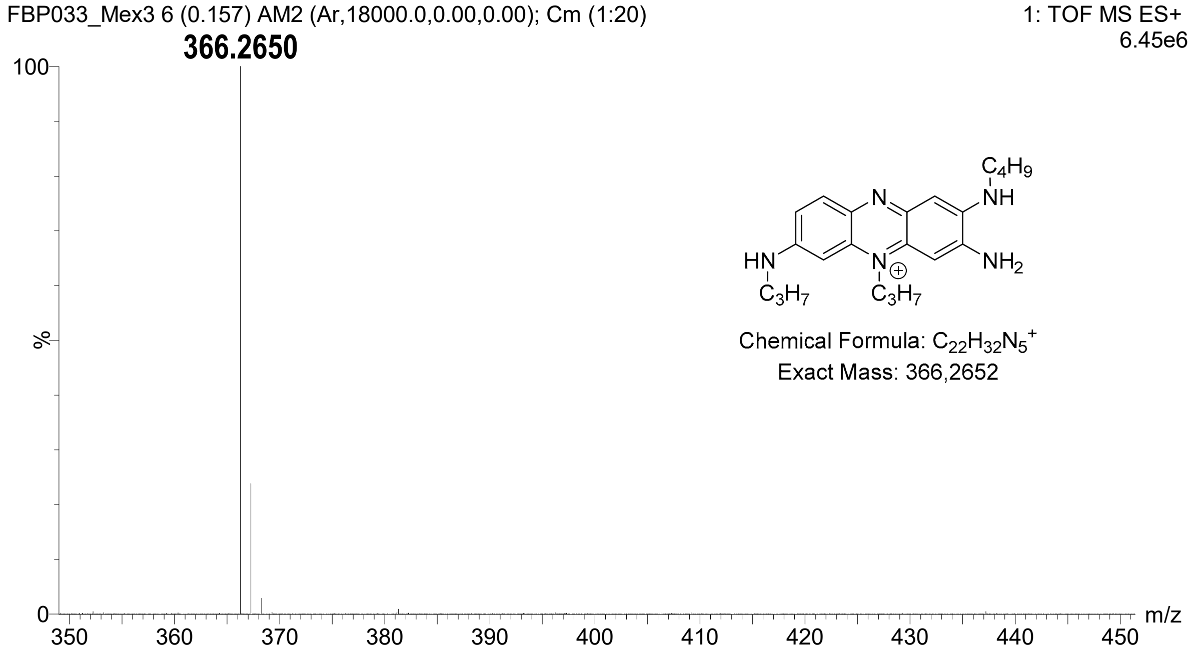

Figure S 30 HRMS spectrum of **5**

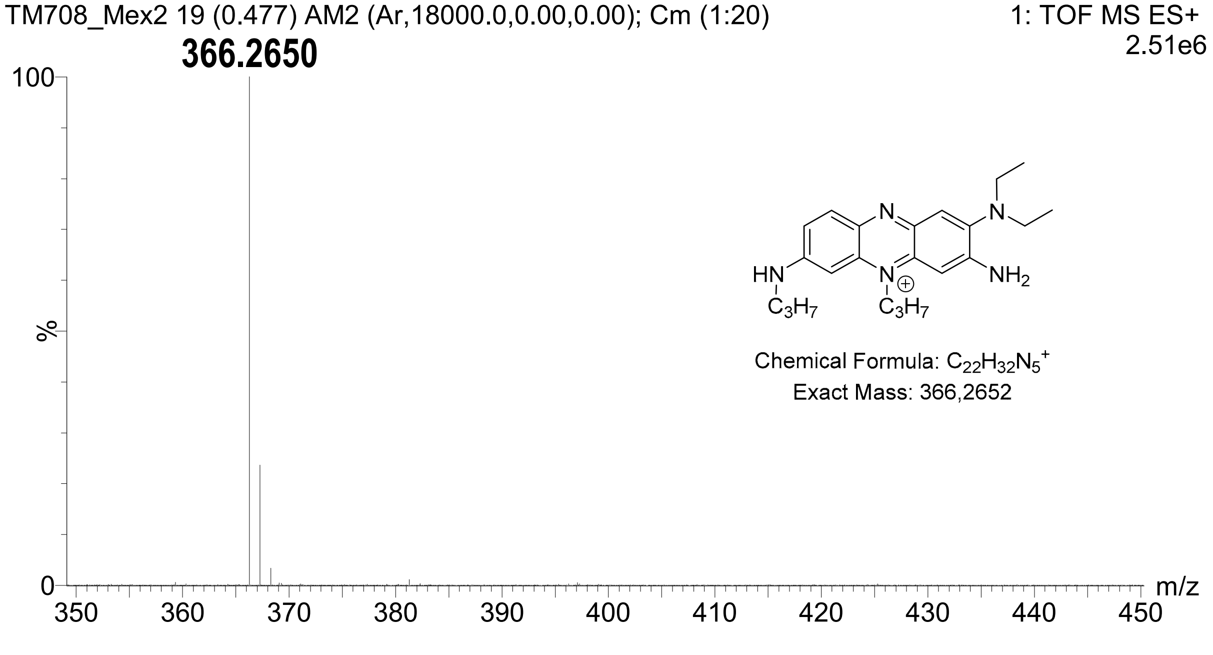

Figure S 31 HRMS spectrum of **6**

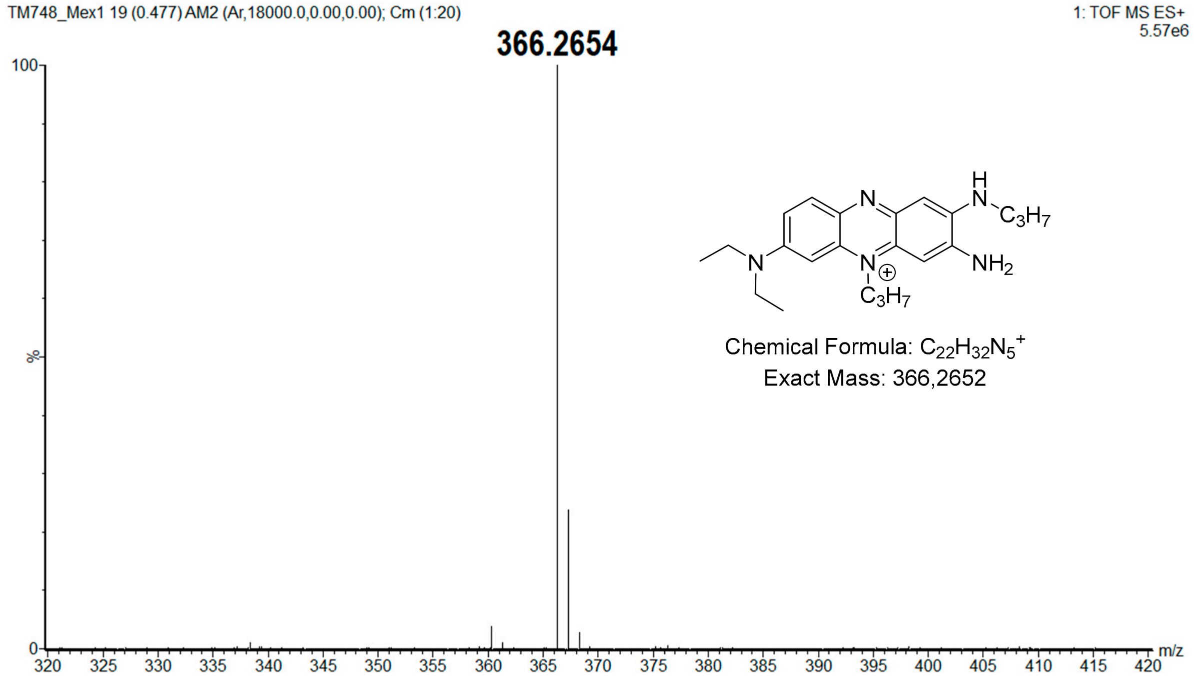

Figure S 32 HRMS spectrum of **7**

III. X-RAY DIFFRACTION

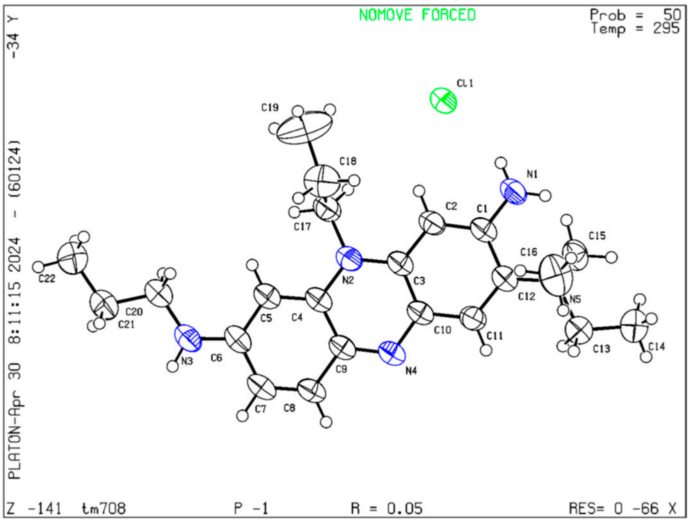

Table S 1 Crystal data and structure refinement for **6**.

Identification code	tm708
Empirical formula	$C_{22}H_{32}ClN_5$
Formula weight	401.97
Temperature/K	295
Crystal system	triclinic
Space group	P-1
a/Å	9.9695(3)
b/Å	10.4033(3)
c/Å	11.6769(3)
α/°	66.444(3)
β/°	89.633(2)
γ/°	88.477(3)
Volume/Å^3	1109.75(6)
Z	2
ρ_{calc}g/cm^3	1.203
μ/mm^{-1}	1.641
F(000)	432.0
Crystal size/mm^3	0.24 × 0.16 × 0.07
Radiation	Cu Kα (λ = 1.54184)
2Θ range for data collection/°	8.26 to 140.64
Index ranges	$-12 \leq h \leq 12$, $-11 \leq k \leq 12$, $-14 \leq l \leq 14$
Reflections collected	14938
Independent reflections	4185 [R_{int} = 0.0250, R_{sigma} = 0.0183]
Data/restraints/parameters	4185/6/257
Goodness-of-fit on F^2	1.072
Final R indexes [I>=2σ (I)]	R_1 = 0.0520, wR_2 = 0.1565
Final R indexes [all data]	R_1 = 0.0557, wR_2 = 0.1616
Largest diff. peak/hole / e Å^{-3}	0.57/-0.30

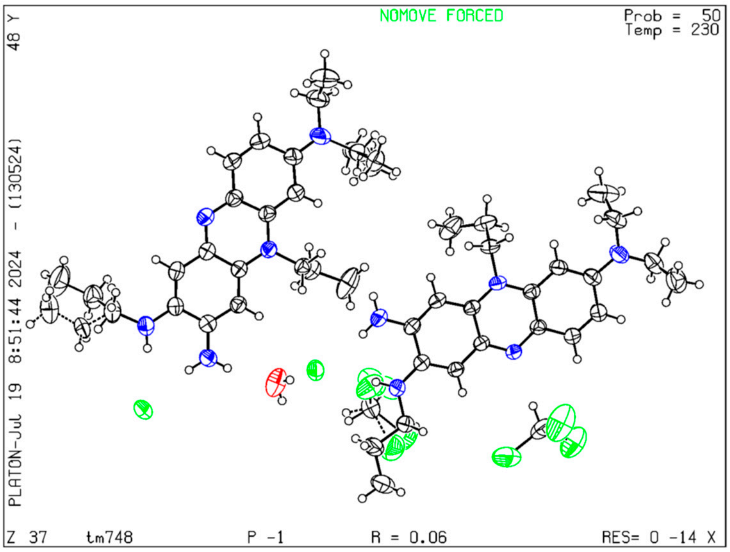

Table S 2 Crystal data and structure refinement for **7**.	
Identification code	tm748
Empirical formula	$C_{46}H_{68}Cl_8N_{10}O$
Formula weight	1060.70
Temperature/K	230.00(10)
Crystal system	triclinic
Space group	P-1
a/Å	12.1257(3)
b/Å	14.3384(3)
c/Å	18.1838(4)
α/°	106.130(2)
β/°	99.210(2)
γ/°	108.995(2)
Volume/Å^3	2760.31(12)
Z	2
ρ_{calc}g/cm^3	1.276
μ/mm^{-1}	4.067
F(000)	1116.0
Crystal size/mm^3	0.18 × 0.18 × 0.16
Radiation	Cu Kα (λ = 1.54184)
2Θ range for data collection/°	6.97 to 145.12
Index ranges	$-15 \leq h \leq 14$, $-17 \leq k \leq 17$, $-14 \leq l \leq 22$
Reflections collected	24344
Independent reflections	10688 [R_{int} = 0.0185, R_{sigma} = 0.0195]
Data/restraints/parameters	10688/1/662
Goodness-of-fit on F^2	1.065
Final R indexes [I>=2σ (I)]	R_1 = 0.0583, wR_2 = 0.1646
Final R indexes [all data]	R_1 = 0.0646, wR_2 = 0.1718
Largest diff. peak/hole / e Å^{-3}	0.77/-0.65

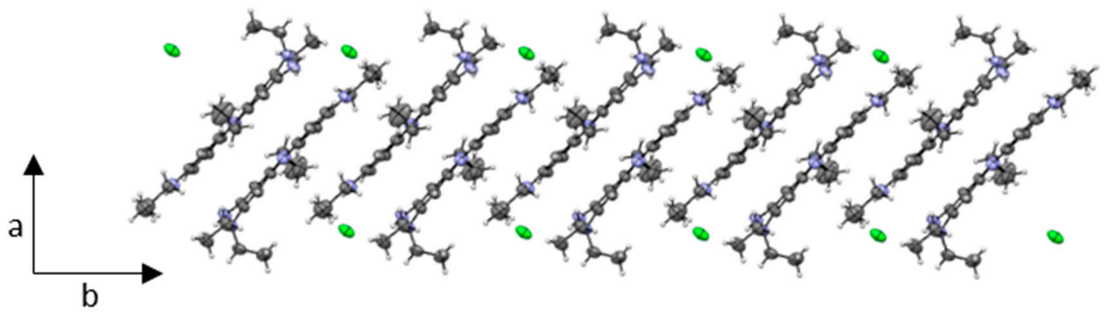

Figure S 33 Molecular packing motif for compound **6**

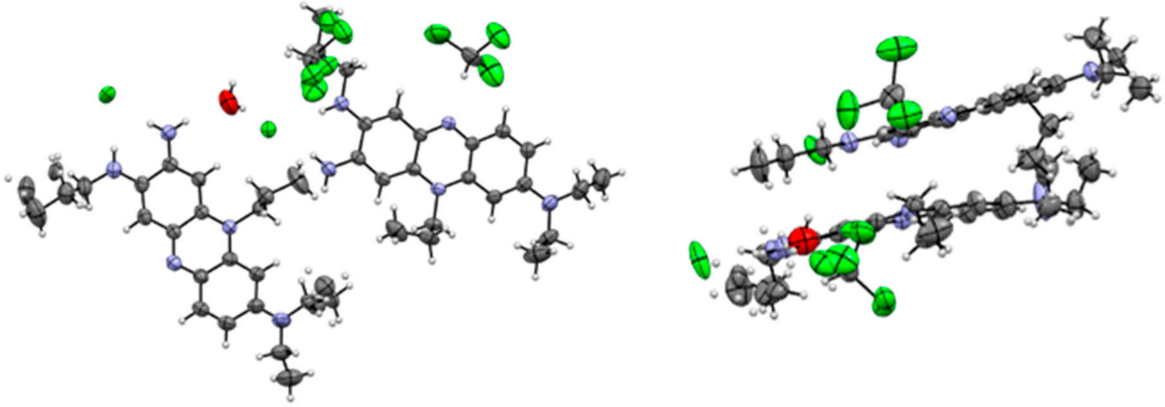

Figure S 34 Asymmetric crystallization unit of **7** composed of two independent molecules

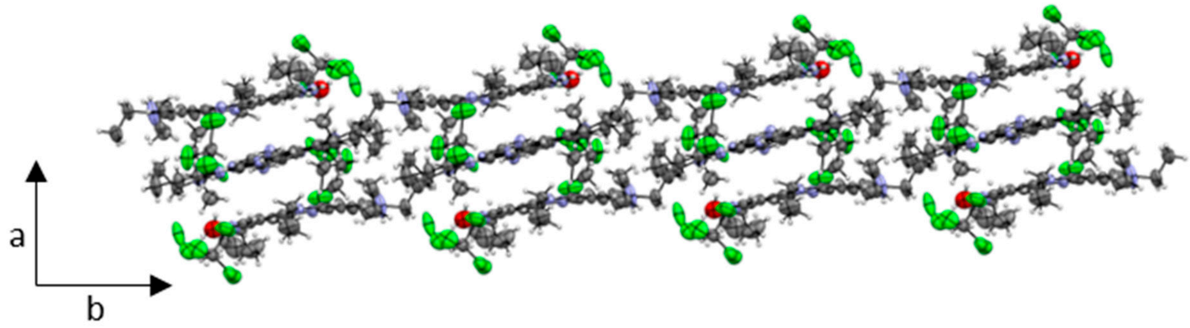

Figure S 35 Molecular packing motif for compound **7**

IV. ADDITIONAL OPTICAL SPECTRA

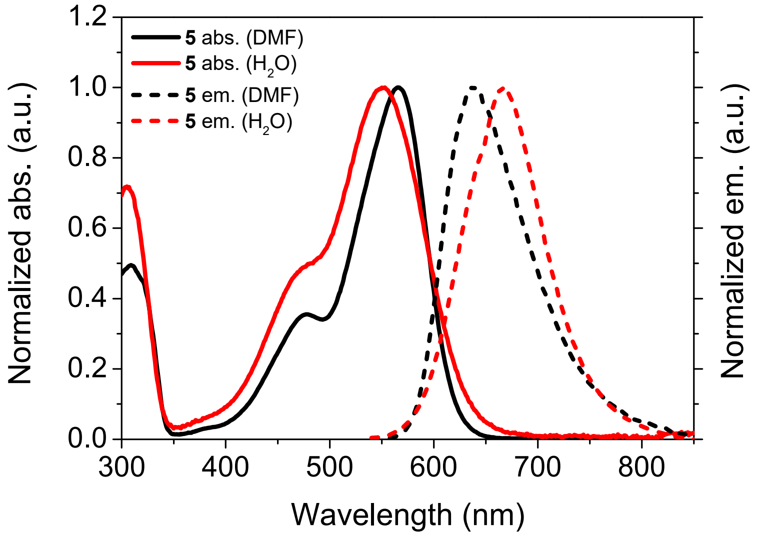

Figure S 36 Normalized electronic absorption (solid line) and emission spectra (dash line) of **5** in DMF and H_2O (λ_{ex} = 530 nm)

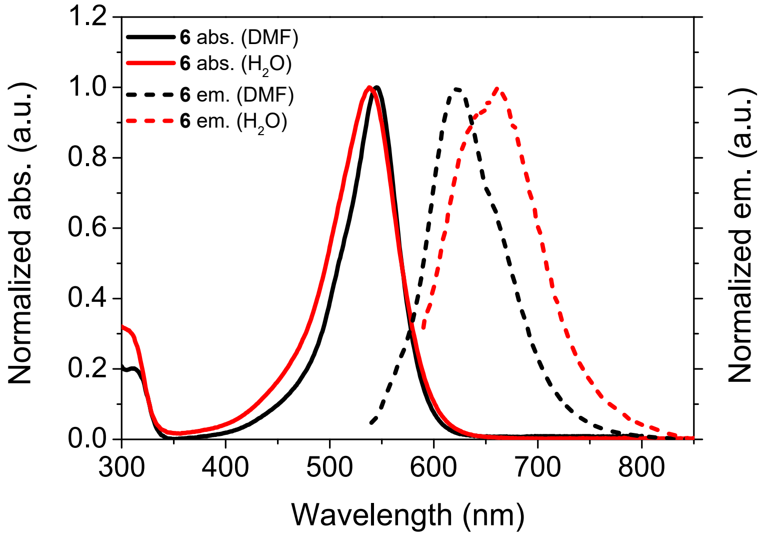

Figure S 37 Normalized electronic absorption (solid line) and emission spectra (dash line) of **6** in DMF (λ_{ex} = 530 nm) and H_2O (λ_{ex} = 565 nm)

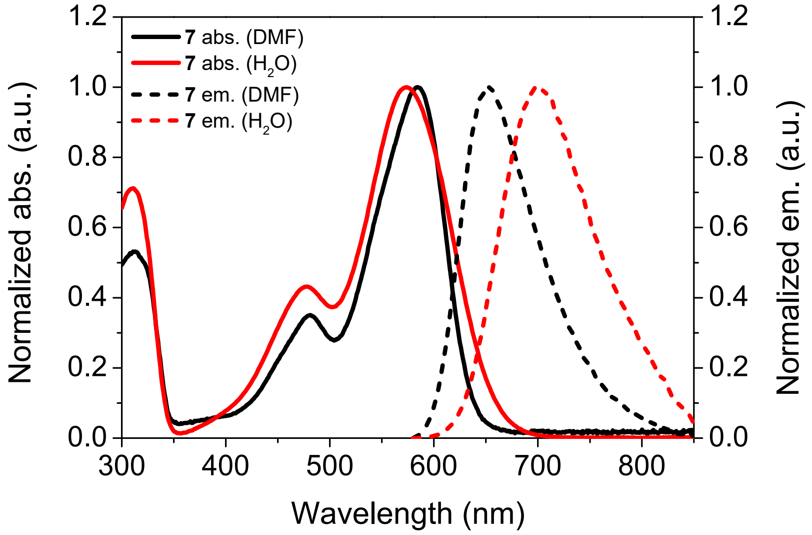

Figure S 38 Normalized electronic absorption (solid line) and emission spectra (dash line) of **7** in DMF and H_2O (λex = 565 nm)

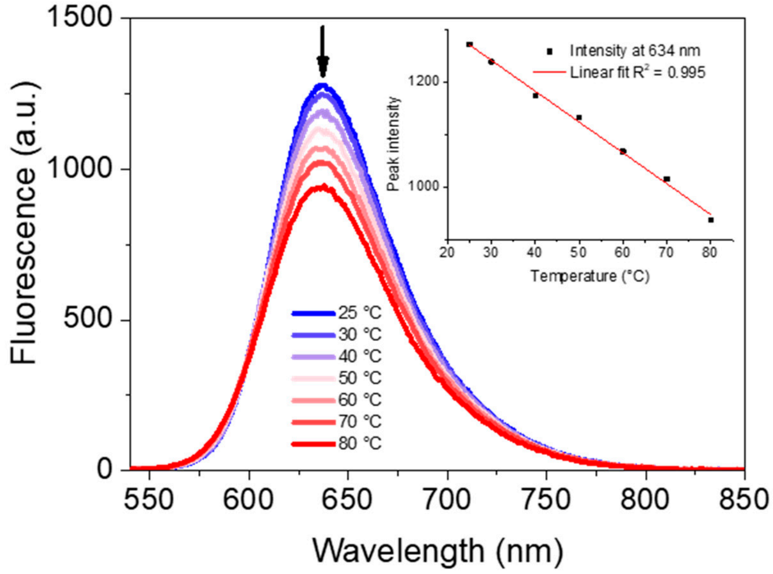

Figure S 39 Fluorescence spectra of **5** in DMSO under gradual heating from 25 °C to 80 °C, λ_{ex} = 530 nm. The inset shows the linear relationship between the intensity of the emission (taken as the maximum value of the peak) and temperature.

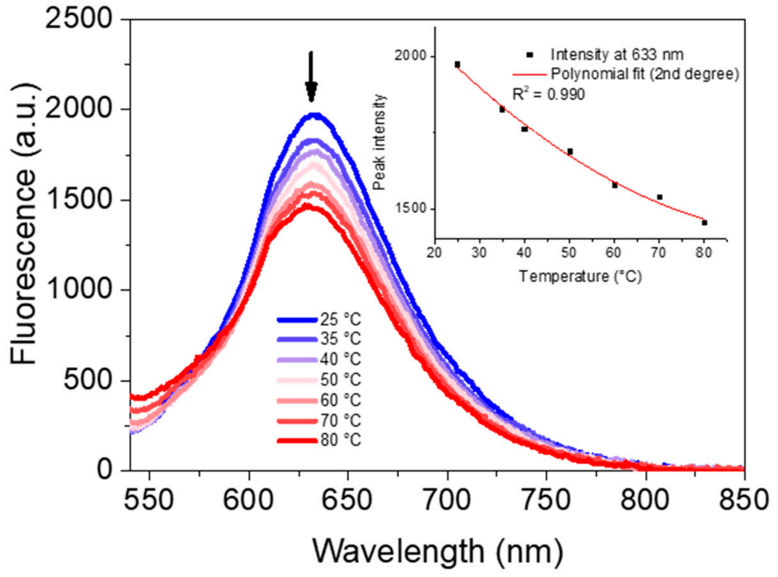

Figure S 40 Fluorescence spectra of **6** in DMSO under gradual heating from 25 °C to 80 °C, λ_{ex} = 515 nm. The inset shows the relationship between the intensity of the emission (taken as the maximum value of the peak) and temperature which fits a second-degree polynomial equation.

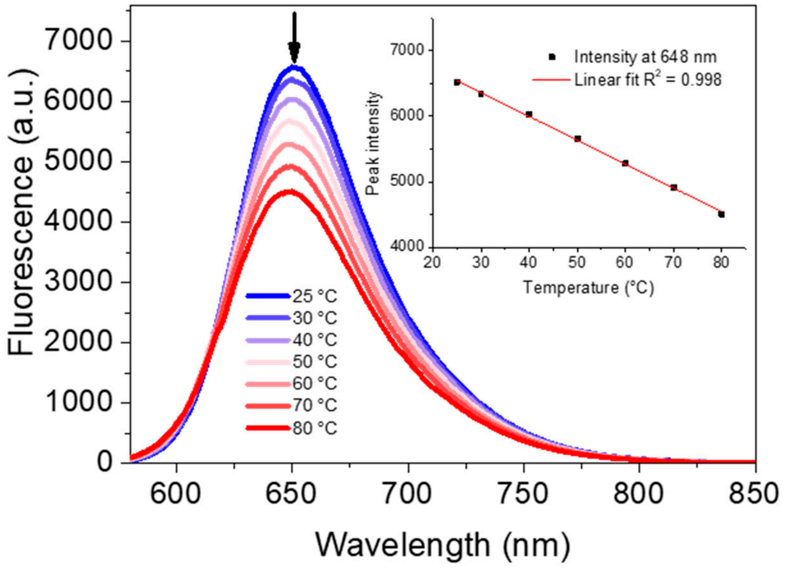

Figure S 41 Fluorescence spectra of **7** in DMSO under gradual heating from 25 °C to 80 °C, λ_{ex} = 565 nm. The inset shows the linear relationship between the intensity of the emission (taken as the maximum value of the peak) and temperature.

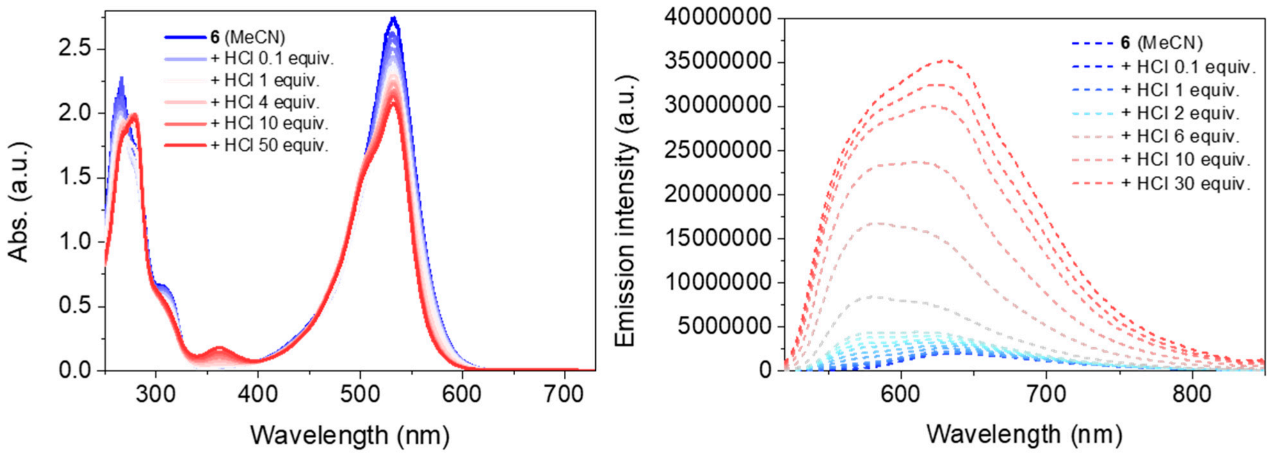

Figure S 42 Electronic absorption (left, solid lines) and emission (right, dotted lines) spectra of **6** in acetonitrile with the gradual addition of HCl. Working concentrations: 4.9131 x 10^{-5} M for absorption and 2.4565 x 10^{-6} M for emission; emission recorded by excitation at 510 nm

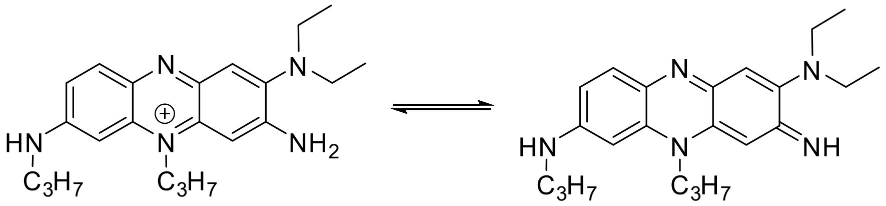

6
emissive

[6-H⁺]
poorly emissive

Scheme S 1 Structure of the emissive cationic and poorly-emissive neutral form of **6**
